# An integrative transcriptome analysis reveals potential predictive, prognostic biomarkers and therapeutic targets in colorectal cancer

**DOI:** 10.1186/s12885-022-09931-4

**Published:** 2022-07-30

**Authors:** Pouria Samadi, Meysam Soleimani, Fatemeh Nouri, Fatemeh Rahbarizadeh, Rezvan Najafi, Akram Jalali

**Affiliations:** 1 Research Center for Molecular Medicine, Hamadan University of Medical Sciences, Hamadan, Iran; 2 Department of Pharmaceutical Biotechnology, School of Pharmacy, Hamadan University of Medical Sciences, Hamadan, Iran; 3 Department of Medical Biotechnology, Faculty of Medical Sciences, Tarbiat Modares University, Tehran, Iran

**Keywords:** Colorectal cancer, lncRNA, miRNA, Machine learning, Deep learning, WGCNA, Diagnosis, Systems biology

## Abstract

**Background:**

A deep understanding of potential molecular biomarkers and therapeutic targets related to the progression of colorectal cancer (CRC) from early stages to metastasis remain mostly undone. Moreover, the regulation and crosstalk among different cancer-driving molecules including messenger RNAs (mRNAs), long non-coding RNAs (lncRNAs) and micro-RNAs (miRNAs) in the transition from stage I to stage IV remain to be clarified, which is the aim of this study.

**Methods:**

We carried out two separate differential expression analyses for two different sets of samples (stage-specific samples and tumor/normal samples). Then, by the means of robust dataset analysis we identified distinct lists of differently expressed genes (DEGs) for Robust Rank Aggregation (RRA) and weighted gene co-expression network analysis (WGCNA). Then, comprehensive computational systems biology analyses including mRNA-miRNA-lncRNA regulatory network, survival analysis and machine learning algorithms were also employed to achieve the aim of this study. Finally, we used clinical samples to carry out validation of a potential and novel target in CRC.

**Results:**

We have identified the most significant stage-specific DEGs by combining distinct results from RRA and WGCNA. After finding stage-specific DEGs, a total number of 37 DEGs were identified to be conserved across all stages of CRC (conserved DEGs). We also found DE-miRNAs and DE-lncRNAs highly associated to these conserved DEGs. Our systems biology approach led to the identification of several potential therapeutic targets, predictive and prognostic biomarkers, of which lncRNA LINC00974 shown as an important and novel biomarker.

**Conclusions:**

Findings of the present study provide new insight into CRC pathogenesis across all stages, and suggests future assessment of the functional role of lncRNA LINC00974 in the development of CRC.

**Supplementary Information:**

The online version contains supplementary material available at 10.1186/s12885-022-09931-4.

## Introduction

Colorectal cancer (CRC) is one of the most diagnosed cancers of the gastrointestinal tract around the world. There were an estimated 147,950 new cases and 53,200 deaths related to CRC in the United States in 2020 [1]. The CRC burden can be significantly decreased with early detection at precancerous-stage adenoma, which has been demonstrated to be effective in alleviating the incidence of CRC, and reducing economic burden [2]. The average five-year survival rate of CRC declines sharply from 90 to 40% in the progression from early-stage adenoma into malignant cancer [3]. In this regard, the correlation between disease survival and the stage at diagnosis, provides insights into how therapy outcomes differ depending on extent of tumor spread. Currently, there are no reliable biomarkers that can predict adenoma to carcinoma sequence. In order to find potential diagnostic, predictive, prognostic, as well as therapeutic biomarkers, understanding the molecular pathogenesis underlying early-stage adenoma progression into malignant forms is needed [4].

The development of high-throughput technologies and computational biology advancements have led to the identification of novel biomarkers and therapeutic targets in CRC [5]. However, different microarray and next-generation RNA sequencing (RNA-seq) platforms and small sample sizes in research has led to high variabilities and poor statistical inferences among studies [6]. In this regard, the widely used Robust Rank Aggregation (RRA) method can integrate differentially expressed gene (DEG) lists of different datasets, thereby greatly reduce biases between multiple data sets and overcoming the challenges caused by small sample sizes [7]. In addition, by using weighted gene co-expression network analysis (WGCNA) as a powerful systems biology method we can identify potential patterns of gene associations (as modules) between different samples based on the interconnectivity of gene sets. Moreover, by providing a robust network-based strategy, WGCNA analyses the network density and topology pattern of modules to identify some modules and their highly connected genes (hub genes) that are not preserved between normal and disease samples and have significant relationship with clinical trait of interest [8]. These modules may potentially be involved in the biological processes of interest clinical trait and be used as candidate biomarkers or therapeutic targets [9].

Micro-RNAs (miRNAs) and long non-coding RNAs (lncRNAs) playing key roles in various human diseases, particularly in cancer, through regulating messenger RNA (mRNA) stability and translation [10]. These dysregulated non-coding RNAs (ncRNAs) have been shown to significantly affect different hallmarks of cancer such as cell growth and proliferation, apoptosis, cell cycle regulation, invasion, metastasis, drug resistance, etc. Moreover, both miRNAs and lncRNAs may act as tumor suppressors or oncogenes to regulate their targeting mRNAs in various cancers especially in CRC [11]. In this regard, by using the expression profiles of ncRNAs and mRNAs, we have explored important interactions of miRNA-mRNA, lncRNA-mRNA, and miRNA-lncRNA, to construct the mRNA-lncRNA-miRNA co-regulatory network in CRC. Finally, a potential lncRNA named LINC00974, targeting almost half of hub genes was identified as CRC progression-associated factor, while its functional role in CRC still remains unclear.

Machine learning (ML) and deep learning (DL) as subsets of artificial intelligence (AI) are believed to have a dominant effect in the clinical setting for cancer diagnosis and treatment in near future [12]. Technological breakthroughs in AI and ML have paved the way towards the development of autonomous and accurate disease detection and classification tools at a very early stage specifically in cancer to meet the future challenges [13]. In the last 5 years, ML and DL have been increasingly used for screening, diagnosis, and treatment of CRC based on different features, such as histopathological images [14–16], drug sensitivity [17], immunological profile [18], as well as integration of clinical, therapeutic, and laboratory information. However, employment of biomarker-based ML model validation in CRC is relatively limited [19]. Furthermore, the quality of input features could reflect the effectiveness of prediction based on ML models. Therefore, in this study, we integrated ML model validation for further validation of the key genes and candidate lncRNAs obtained from mRNA-lncRNA-miRNA co-regulatory network.

Taken together, the present study was designed with the following points to achieve the final goal: first, we defined the combination of significant DEGs determined by RRA and WGCNA in each stage of CRC (stage I to IV) as stage-specific genes. Moreover, the intersection between RRA and WGCNA was considered as conserved hub genes across all CRC stages. Second, we further explored the biological significance of stage-specific genes and hub genes in the downstream processes to identify potential biomarkers for diagnosis (using ML and DL), prognosis (using survival analysis) and therapeutic targets (using protein–protein interaction (PPI) and non-coding RNA regulatory networks). Third, the candidate hub gene, lncRNA LINC00974 was further evaluated and experimentally validated, to assess its biological significance in CRC carcinogenesis.

## Materials and methods

### Study design and data collection

**Fig. 1:**
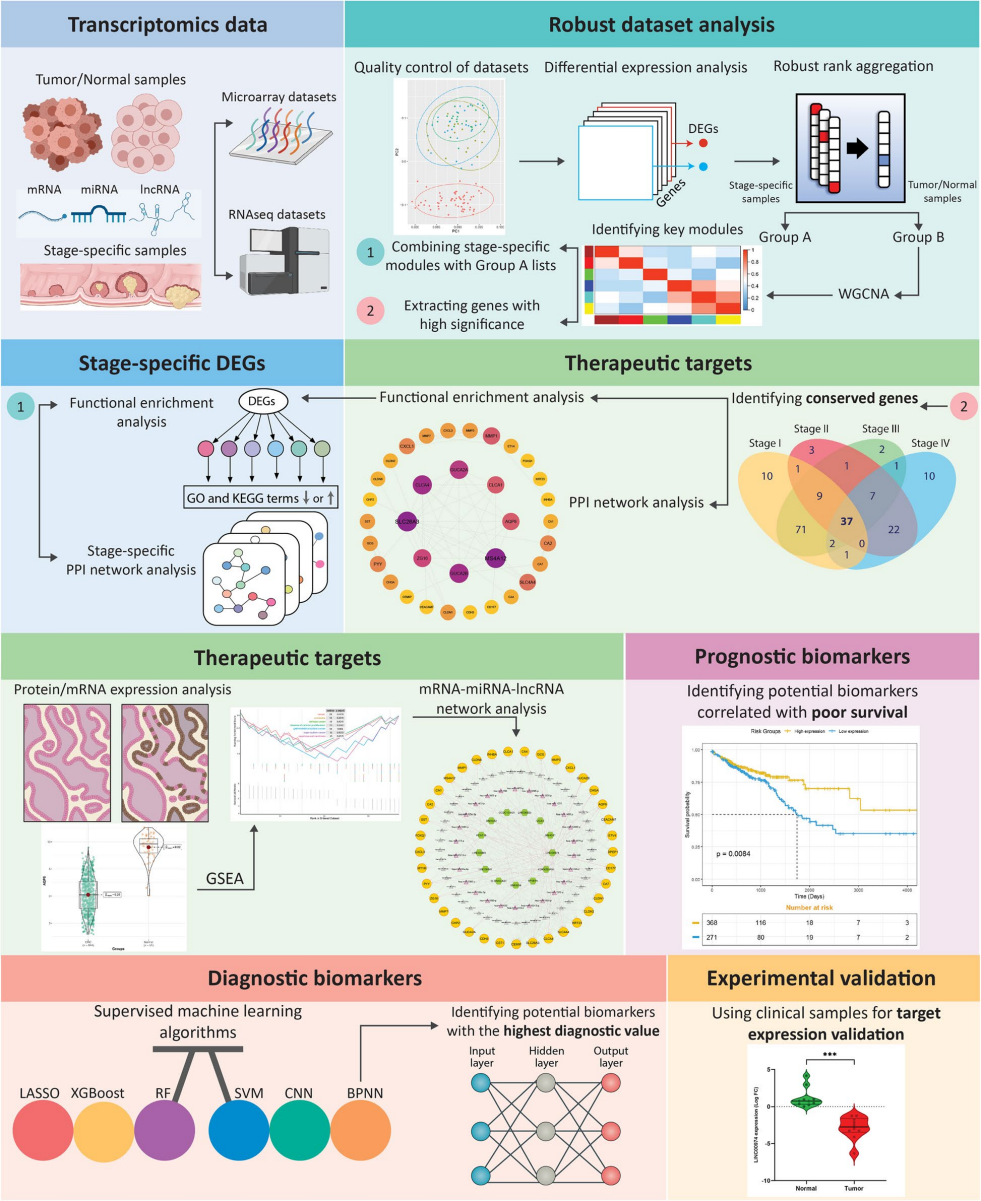
Two different sets of microarray and next-generation sequencing data were downloaded and analyzed for identifying differentially expressed mRNAs (DEGs), miRNAs (DE miRNAs), and lncRNAs (DE-lncRNAs) in CRC and normal tissues. Next, DEGs were imported to RRA to find the most significant DEGs across all datasets and obtain distinct lists as Group A and Group B. DEGs in the Group B were then used for WGCNA to determine key modules related to each stage of CRC, separately. By combining DEGs in the Group A and B, stage-specific DEGs were found and investigated for protein-protein interaction (PPI) network and functional enrichment analysis. Furthermore, by setting two different cut-off criteria and selecting common DEGs, conserved hub genes across all stages were also obtained. By using DE-miRNAs and DE-lncRNAs as well as conserved hub genes, mRNA-miRNA-lncRNA regulatory network was constructed to find potential therapeutic targets. Ultimately, a comprehensive downstream analysis was performed to identify potential prognostic and diagnostic biomarkers, through different approaches

Flow chart of the present study is shown in Fig. 1. Gene expression data were obtained from the GEO database (http://www.ncbi.nlm.nih.gov/geo/) up to November 25, 2021; we screened GEO microarray datasets with regarding to the following criteria: (1) gene expression data from CRC and adjacent normal tissue samples were evaluated; (2) datasets contained a minimum of five tumor and adjacent normal tissue samples; 3) the number of genes in a single dataset was > 10,000. After careful consideration, we have divided GEO datasets into Group A and Group B to identify robust DEGs based on two distinct powerful methods of RRA and WGCNA, respectively. In this regard, GEO datasets for stage-specific analysis of DEGs using RRA (Group A) were divided into four groups (Table 1), including stage I (five datasets), stage II (six datasets), stage III (six datasets) and stage IV (four datasets). On the other side, for WGCNA, as another robust algorithm to find significant DEGs related to a specific clinical trait (in this study, different CRC stages), a total number of ten datasets containing tumor/normal samples (Table 2) were analyzed (Group B). Additionally, we downloaded the RNA-seq data and clinical information containing 644 CRC tissues and 51 normal tissues for COAD (colon adenocarcinoma) and READ (rectal adenocarcinoma) from the TCGA database (https://portal.gdc.cancer.gov/), and utilized them in the study. Therefore, by adopting RRA and WGCNA, we believe that identifying stage-specific genes would be more robust and reliable. The detailed information of the datasets used in this study is shown in Tables 1 and 2.

**Table 1:** Characteristics of the included datasets for Group A in the present study

TNM Staging	Groups	# Samples (Cases/Control)	Platform	Genes	DEGs (UR/DR)
Stage I	GSE35602	13 (5/8)	GPL6480	41,108	3635 (2629/1006)
	GSE41258	81 (27/54)	GPL96	22,283	924 (366/558)
	GSE89076	42 (8/34)	GPL16699	62,976	4953 (2649/2304)
	GSE117606	74 (15/59)	GPL25373	19,276	424 (156/268)
	GSE71187	25 (13/12)	GPL6480	41,108	3784 (1535/2249)
	TCGA-COAD	125 (84/41)	Illumina	56,499	4520 (1631/2889)
	TCGA-READ	41 (31/10)	Illumina	56,493	4918 (1640/3278)
Stage II	GSE35602	16 (8/8)	GPL6480	41,108	4427 (2542/1885)
	GSE41258	103 (49/54)	GPL96	22,283	808 (281/527)
	GSE89076	42 (8/34)	GPL16699	62,976	4606 (2596/2010)
	GSE117606	72 (23/59)	GPL25373	19,276	562 (232/329)
	GSE71187	29 (17/12)	GPL6480	41,108	3656 (1384/2272)
	TCGA-COAD	132 (191/41)	Illumina	56,499	4660 (1684/2976)
	TCGA-READ	64 (54/10)	Illumina	56,493	4694 (1580/3114)
Stage III	GSE35602	18 (10/8)	GPL6480	54,675	2918 (2047/871)
	GSE41258	102 (48/54)	GPL96	22,283	800 (330/470)
	GSE83889	128 (93/35)	GPL10558	48,107	1501 (922/579)
	GSE89076	42 (11/34)	GPL16699	62,976	4955 (2787/2168)
	GSE117606	73 (24/59)	GPL25373	19,276	477 (184/293)
	GSE71187	63 (51/12)	GPL6480	41,108	3363 (1268/2095)
	TCGA-COAD	176 (135/41)	Illumina	56,499	4562 (1614/2948)
	TCGA-READ	64 (54/10)	Illumina	56,493	4863 (1763/3100)
Stage IV	GSE41258	109 (55/54)	GPL96	22,283	796 (297/499)
	GSE89076	38 (6/34)	GPL16699	62,976	4798 (2460/2338)
	GSE71187	18 (6/12)	GPL6480	41,108	4584 (1803/2781)
	GSE49355	36 (20/16)	GPL96	22,283	814 (357/457)
	TCGA-COAD	109 (68/41)	Illumina	56,499	4619 (1657/2962)
	TCGA-READ	35 (25/10)	Illumina	56,493	4568 (1844/2724)

**Table 2:** Characteristics of the included datasets for Group B in the present study

Groups	# Samples (Cases/Control)	Platform	Genes	DEGs (UR/DR)
GSE89076	60 (30/30)	GPL16699	58,717	4500 (2279/2221)
GSE71187	111 (99/12)	GPL6480	41,091	3526 (1320/2206)
GSE23878	54 (30/24)	GPL570	54,675	2288 (832/1456)
GSE41657	36 (24/12)	GPL6480	41,076	3284 (1310/1974)
GSE113513	28 (14/14)	GPL15207	49,395	1848 (746/1102)
GSE87211	359 (202/157)	GPL13497	34,127	3170 (1557/1613)
GSE74602	55 (29/26)	GPL6104	22,184	1700 (888/812)
GSE37182	166 (79/87)	GPL6947	48,803	857 (362/495)
GSE110225	28 (14/14)	GPL570	54,675	1908 (860/1048)
GSE37364	61 (24/37)	GPL570	54,675	3476 (1839/1637)
TCGA-COAD	519 (478/41)	Illumina	56,499	5580 (3595/1985)
TCGA-READ	176 (166/10)	Illumina	56,493	5604 (3469/2135)

### Pre-processing and differential expression analysis

The quality control of the normalized datasets was performed using principal component analysis (PCA) to identify outlier samples without biological relevance and removed them prior to DEG analysis. To identify the statistically significant DEGs between CRC and adjacent normal tissue samples in GEO microarray datasets, the R (version 4.1.0) package limma was used [20]. Moreover, for differential expression analysis of RNA-seq data (HTSeq-Counts), TCGA-COAD and TCGA-READ samples were retrieved from GDC data using TCGAbiolinks [21] and batch-corrected by ComBat package in Bioconductor. For filtering and identification of the significant DEGs, edgeR [22] and limma packages were employed. In this regard, the lowly expressed genes were removed using the edgeR package. To remove composition biases between the libraries the trimmed mean of m-values (TMM) normalization method was used. The variance modeling at the observational level (VOOM) function from the limma package was also applied to convert the read counts into logCPMs (log Counts per million). The statistically significant DEGs for both GEO microarray and RNA-Seq TCGA datasets were filtered based on the cut-off criteria as false discovery rate threshold (FDR) < 0.05 and |log2-fold change (FC)| > 1.

### Identification of robust DEGs

To integrate different lists of DEGs into a robust final list of DEGs for each group, we applied the RRA algorithm (R package). In this case, results from each dataset were ranked according to the FC value of each gene and RRA algorithm looked at how the DEGs was ranked in the input lists and compared this to the baseline [7]. Finally, for identification of the robust DEGs, the RRA analysis was implemented under an FDR < 0.05 cutoff point.

### WGCNA and identification of the key modules

WGCNA algorithm was employed to construct a co-expression network to identify modules associated with stage I to IV using R package WGCNA [8]. Since the input data of WGCNA is usually less than 5000 genes, we used the preliminary screened genes resulted from RRA (Group B) with expression data analyzed with DESeq2 package [23] (as recommended by WGCNA authors) and retrieved from the largest sample size (TCGA COAD-READ). In this regard, Pearson’s correlations matrices were applied between all pair-wise genes to generate the adjacency matrix. Next, the soft threshold power of β = 4 (scale free R^2^ = 0.9) was used to achieve an adjacency matrix with scale-free topology and reduced noise of the correlations. The adjacency matrix was then transformed into a topological overlap matrix (TOM), based on the TOM-based dissimilarity measure with a minimum module size of 30 and cut height of 0.4 to merge similar modules. For determination of key modules, average linkage hierarchical clustering was employed to classify robust DEGs with similar expression into the same gene module. The correlation between module eigengenes (MEs) and clinical traits (stage I to IV) was calculated to identify clinically significant modules. Finally, these modules with the highest correlation with clinical traits were selected to explore their biological function. Hub genes with a module membership (MM) > 0.7 were also selected for downstream analysis [24].

### Identification of stage-specific and conserved hub genes

In order to determine hub genes associated with a specific stage, DEGs resulted from WGCNA modules and RRA were combined for each stage, separately. Moreover, to find the most important cancer-driving genes, filtering was applied in accordance with the following criteria: 1) top 30 downregulated and upregulated DEGs (based on the log2FC) from Group A and 2) DEGs with a MM > 0.7 from Group B. Finally, VennDiagram package in r was used to find these conserved DEGs across all stages of CRC [25].

### Functional enrichment analysis

The Gene Ontology (GO) and Kyoto Encyclopedia of Genes and Genomes (KEGG) (www.kegg.jp/kegg/kegg1.html) [26] enrichment analysis of stage specific and conserved DEGs was performed by R packages ClusterProfiler and GOplot, respectively [27]. ClusterProfiler results were displayed in the dotplot, wherein enriched pathways were presented using gene ratio, adjusted *p*-value, and count. Moreover, chord plots depicted the relationship between conserved DEGs and GO terms (biological process, cellular component, molecular function) and KEGG pathways. Terms with adjusted *p* < 0.05 were considered statistically significant.

### PPI and construction of lncRNA–miRNA–mRNA regulatory networks for identifying therapeutic targets

After the identification of stage-specific and conserved core genes, PPI network information was provided using Search Tool for the Retrieval of Interacting Genes (STRING) database (https://string-db.org/cgi/input.pl/) [28]. DEGs with the high confidence score (combined score > 0.7) from all active sources such as experiments, databases, co-expressions and etc. were imported into Cytoscape 3.8.2 for network construction and visualization. Then, for defining stage-specific hub genes, combined DEGs from each stage were processed for module analysis, using Molecular Complex Detection (MCODE) clustering algorithm in Cytoscape software with default parameters. In regard to the smaller number of conserved hub genes, DEGs with the combined score > 0.4 were placed for MCODE analysis, to identify the most important DEGs with high degrees or MCODE scores ≥7 as therapeutic targets.

Prior to construct lncRNA–miRNA–mRNA regulatory network, we identified DE-miRNAs and DE-lncRNAs from TCGA COAD-READ by implementing edgeR/LIMMA method and setting a FDR < 0.05 and |log2(FC)| > 1 cut-off as described earlier. Moreover, we omitted pseudogenes lncRNAs based on the manual annotation. In order to find miRNA-mRNA, lncRNA-mRNA, and miRNA-lncRNA interactions for the construction of regulatory network, five databases as following were used: miRWalk (http://mirwalk.umm.uni-heidelberg.de/), miRabel (http://bioinfo.univ-rouen.fr, which uses data from MiRanda, Pita, SVmicro and TargetScan prediction tools), miRDB (http://www.mirdb.org/), MiRTarBase (https://mirtarbase.cuhk.edu.cn), and miRcode (http://www.mircode.org/, specifically for miRNA-lncRNA interaction). Furthermore, for mRNA-lncRNA interaction, the Pearson’s correlation between DE-lncRNAs and DEGs were calculated. Those pairs with the Pearson’s correlation coefficients (PCCs) > 0.6 and FDR < 0.05 were considered as strong interactions. Finally, mRNAs, miRNAs, and lncRNAs pairs that have differential expression were chose to construct final network.

### Visualization of gene expression patterns and chromosome locations

Circos display of expression patterns (from RRA) and chromosomal locations of conserved hub genes was presented using the shinyCircos package in R [29]. Moreover, using Pearson correlation in R, DEGs with an average expression correlation > 0.8 have determined to be presented in the core of the Circos plot.

### Validation of hub Gene’s expression levels

The expression data from TCGA COAD-READ, was used to assess expression of conserved hub genes between different AJCC stages and CRC vs normal condition. The violin plots were drawn using R package “ggstatsplot” [30]. Furthermore, to elucidate the differential expression of hub genes at a protein level (immunohistochemistry), the immunohistochemistry images from Human Protein Atlas (HPA) online database (http://www.proteinatlas.org/) were used to discriminate between normal and CRC tumor tissues.

### Gene set enrichment analysis (GSEA)

GSEA is a widely used tool, capable of identifying the biological functions of DEGs based on the differential expression of different gene sets. In this case, we have used ClusterProfiler package, to conduct GSEA for hub genes and lncRNA co-expressed mRNAs on the basis of REACTOME pathway database and with expression data from TCGA COAD-READ and by setting the FDR cutoff to < 0.05.

### Survival analysis of hub genes to identify potential prognostic biomarkers

To determine DEGs and DE-ncRNAs with significant prognostic values, disease-free survival (DFS) and overall survival (OS) analyses were performed. In this regard, for OS analysis a total of 644 TCGA COAD-READ samples were divided into high- and low-expression groups based on best cutoff points calculated by the R package ‘survminer’. The Kaplan-Meier survival analysis with the log-rank test was then performed to identify OS-related genes using the “survival” package and setting a cut-off point of FDR < 0.01. Moreover, for DFS analysis, online resource KM plotter was used to calculate the hazard ratio (HR), with 95% confidence intervals (CI), and the log-rank *p*-value on the webpage after ≤120 months follow-up.

### Machine learning validation to identify potential diagnostic biomarkers

To identify potential novel diagnostic biomarkers among mRNAs, lncRNAs and miRNAs, ML and DL models were applied. First, LASSO (least absolute shrinkage and selection operator) algorithm (in python) was performed on the core genes, with the expression data retrieved from TCGA COAD-READ with the largest sample size, for ranking-based selection of features (genes) based on their weights. Then, six different ML and DL classification models were employed to build the predictive model, with the aim to fit the proper model weights to predict whether the testing sample (genes) belongs to the CRC sample or normal sample. The main parameters of the different ML and DL models were as follows: 1) Logistic LASSO regression with L1-regularization has been used for feature extraction, the penalty coefficient C was set to 0.14, and the optimization algorithm was the “liblinear” method; 2) the penalty parameter C in the support vector machine (SVM) model was set to 0.1, and the radial basis function (RBF) was used as the kernel function; 3) random forest (RF) model was constructed using the maximum number of 200 as sub-decision tree classifiers, and the number of features in each sub-tree was up to five; 4) to minimize the risk of overfitting data in eXtreme Gradient Boosting (Xgboost), the maximum size of the tree (max_depth) was set to 4, the penalty coefficient λ (lambda) was set to 10, and the learning rate hyper-parameter was set to 0.001; 5). In the back propagation neural network (BPNN) model the number of hidden nodes to use in a single hidden layer network was the same as the input layer. The Relu function was adopted as the activation function from the input layer to the hidden layer, and the output layer value, which is compressed by the Sigmoid function was considered as the probability value for output. The batch gradient descent algorithm was also applied as the learning method. Moreover, to quantify model errors the cross-entropy loss function was used, and the number of iterations was adjusted to 1000, the learning rate was also set to 0.01). The one-dimensional convolutional neural network (1D-CNN) was used as the CNN model. A CNN model typically consists of three layers: a convolutional layer, a pooling layer, and a fully connected layer, and our model contained two sub-layers layers for each of these layers (conv1,2; pool1,2; and fc1,2). Conv1 and Conv2 contained two and four output channels with kernel sizes of 3 and 5, respectively. We adopt batch normalization for data right after each convolution and before activation by Relu function. Furthermore, during the training of CNN model, the max pooling was used as the pooling method, and the kernel size and stride were adjusted to 2. The data passed through the pool2 layer was considered as the input of the fc1 layer and Relu activation function was utilized for the neurons of fc1 layer. In addition, sigmoid activation function was used to generate probability value from the output of the fc2 layer. Similar to BPNN, the batch gradient descent method was implemented as the training algorithm, and the same adjustments were applied for the number of iterations, learning rate and cross entropy function. For all models, 1000 Monte Carlo cross-validation replications were used to test the robustness and predictive value of each model. Finally, 1000 values of six indicators [sensitivity, specificity, accuracy, negative predictive value (NPV), positive predictive value (PPV), and area under curve (AUC)] were achieved, and the average value was used to evaluate the generalization performance of different ML and DL models. To evaluate the diagnostic accuracy, we drawn the receiver operating characteristic (ROC) curve based on sensitivity [true positive rate (TPR)] and 1– specificity [false positive rate (FPR)], and calculate the AUC value, which was associated with the model performance. LASSO, SVM, and RF models were all integrated in the scikit-learn package (version 0.22.1), Xgboost was implemented by the xgboost package, and the network structure of BPNN and CNN was built in the Pytorch framework (Google Collab). All models were implemented in python (version 3.7.6).

### Patients

A total of 20 CRC samples and their corresponding adjacent non-cancerous tissues were obtained from Iranian patients who were referred to Poursina Hakim Research Institute during 2021–2022, Esfahan, Iran. The study protocol was approved by the ethical committee of Hamadan University of Medical Science (ethical code: IR.UMSHA.REC.1399.562). Patients received no chemotherapy or radiotherapy prior to sample collection. All samples were stored in − 80 °C till downstream expression analyzes.

### Real-time PCR assay

Total RNA was isolated from tumoral and non-tumoral tissues using RNX-Plus kit (CinnaGen, Iran) and converted into complementary DNA (cDNA) using RevertAid First strand cDNA synthesis kit (Thermo Fisher Scientific, USA). Quantitative reverse transcription PCR (RT-qPCR) was performed in duplicate for each sample based on SYBR Green and a LightCycler 96 Real-Time PCR detection system (Roche, USA) according to the manufacturer’s instructions. For qRT-PCR, LINC00974 primers (Sinaclon, Iran) were: 5′-CCAGTTCATCGCACCTTG-3′ (sense) and 5′-TAGCAATACAGTTCTCGTAGC-3′ (antisense); GAPDH primers (Sinaclon, Iran) were: 5′- AAGGCTGTGGGCAAGGTCATC-3′ (sense) and 5′-GCGTCAAAGGTGGAGGAGTGG-3′ (antisense). The 2^-△△Ct^ method was used to calculate the relative gene expression levels of LINC00974 and GAPDH, which were normalized to the corresponding GAPDH mRNA levels. Beside RT-qPCR, we also tended to validate our results using a more clinically homogenous dataset with higher number of samples. In this case, we used a GEO dataset, GSE87211 as an external test-set for validation of LINC00974 expression with 202 and 157 CRC and normal cases, respectively.

### Statistical methods

Statistical analyses were performed using the R programming language and Graphpad Prism version 9.0 (Graphpad Software). The comparison between mean values of normal and CRC tumor tissues of patients, was assessed using the paired t-test. Correlation tests between expressions were evaluated through the calculation of Pearson correlation coefficients. For all statistical tests, ^∗^*P* < 0.05, ^∗∗^*P* < 0.01, ^∗∗∗^*P* < 0.001 were considered as statistically significant.

## Results

### Identification of robust DEGs in the datasets

Based on the aim of our study, the workflow is based on two groups and divided into three main steps (Fig. 1). The first group (Group A) consisted of nine microarray and RNA-seq datasets that compared expression between normal and different stages (I to IV) of CRC samples. These datasets contained a total of 401 (stage I), 458 (stage II), 666 (stage III), and 345 (stage IV) samples for both normal and CRC groups (Table 1). The number of DEGs for each dataset was ranged from ~ 400 to ~ 4900 (Table 1). On the other side, for Group B, there were twelve datasets with normal and CRC (all stages) samples. The total number of samples in these datasets was 1653, with 1189 and 464 samples for CRC and normal, respectively (Table 2). There were also ~ 850 to ~ 5600 DEGs identified in the Group B datasets (Table 2), with the count of UR DEGs being relatively higher than DR DEGs. Furthermore, the quality of the normalized datasets was ascertained using PCA, as an unsupervised analysis, to identify outlier samples without biological significance. In this regard, PCA plots represented a good separation of CRC and normal samples in all datasets of Groups A and B (Additional file 1. S1 and S2). Finally, the RRA package in R was used to perform integration and analysis of Group A and B datasets to obtain robust DEGs with the FDR < 0.05 and logFC > 1. The number of robust DEGs identified in the Group A were 773 (375 UR, 398 DR), 528 (147 UR, 381 DR), 463 (159 UR, 304 DR), and 303 (72 UR, 231 DR) for stage I to IV, respectively. Moreover, for Group B, there were a total of 1399 robust DEGs, out of which 560 were UR, and 839 were DR. As shown in Additional file 1. S3 and S4, the 20 most significant DEGs (UR and DR) were consistently identified among most of the datasets, implicating the robustness of the results.

### Identification of key modules related to stage I to IV by WGCNA

**Fig. 2:**
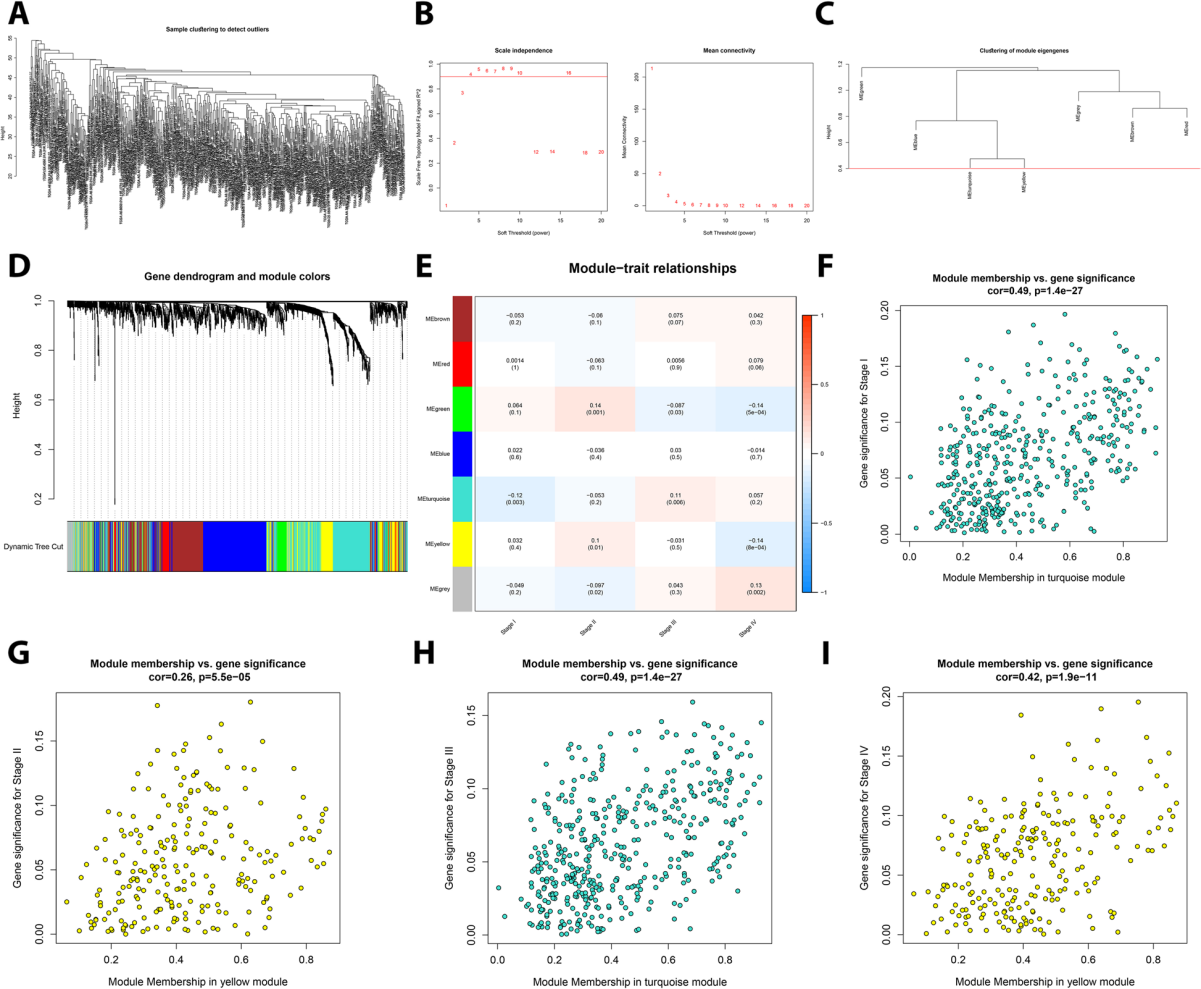
Identification of key modules highly correlated with stage I to IV in the TCGA COAD-READ dataset. **A** The sample clustering tree of CRC samples in the TCGA dataset to find and remove outlier samples. **B** Analysis of scale-free fit index (left) and mean connectivity (right) for each soft-threshold power. **C** Clustering of module eigengenes with the red line representing cut height (0.4). **D** Clustering dendrograms of robust DEGs and related modules based on a dissimilarity measure (1- TOM). **E** Heatmap of the correlation between module eigengenes and stage I to IV (as clinical traits) in CRC. Each cell contains *p*-value and the correlation coefficient. Scatter plot of key modules related to stage I **(F)**, stage II **(G)**, stage III **(H)** and stage IV **(I)**

The total number of 1399 robust DEGs obtained from RRA (Group B) were employed for the construction of co-expression network on the TCGA COAD-READ dataset according to the following steps. Initially, a sample clustering tree was drawn to find and remove outlier samples without biological relevance (Fig. 2A). Next, by setting a soft-thresholding power of β = 4 (scale free R^2^ = 0.9) and cut height as 0.4, a total of seven modules with different colors were identified with non-clustering DEGs shown in gray (Fig. 2B, C, D). Then, by evaluating the module-trait relationships (Fig. 2E), the yellow (434 DEGs) and turquoise (235 DEGs) modules showed greater significance in relation to clinical information based on stage I to IV. In this regard, turquoise module had shown the most correlation and significance with stage I (Cor = 0.49/*p =* 1.4 e^− 27^) and III (Cor = 0.26/*p =* 5.5 e^− 5^), and yellow module was associated with stage II (Cor = 0.49/*p =* 1.4 e^− 27^) and IV (Cor = 0.42/*p =* 1.9 e^− 11^). Finally, based on the MM > 0.7 as a cut-off, we selected 71 and 20 hub genes for turquoise and yellow modules, respectively (Fig. 2F, G, H, I).

### Identification of hub genes and their functional enrichment analysis

**Fig. 3:**
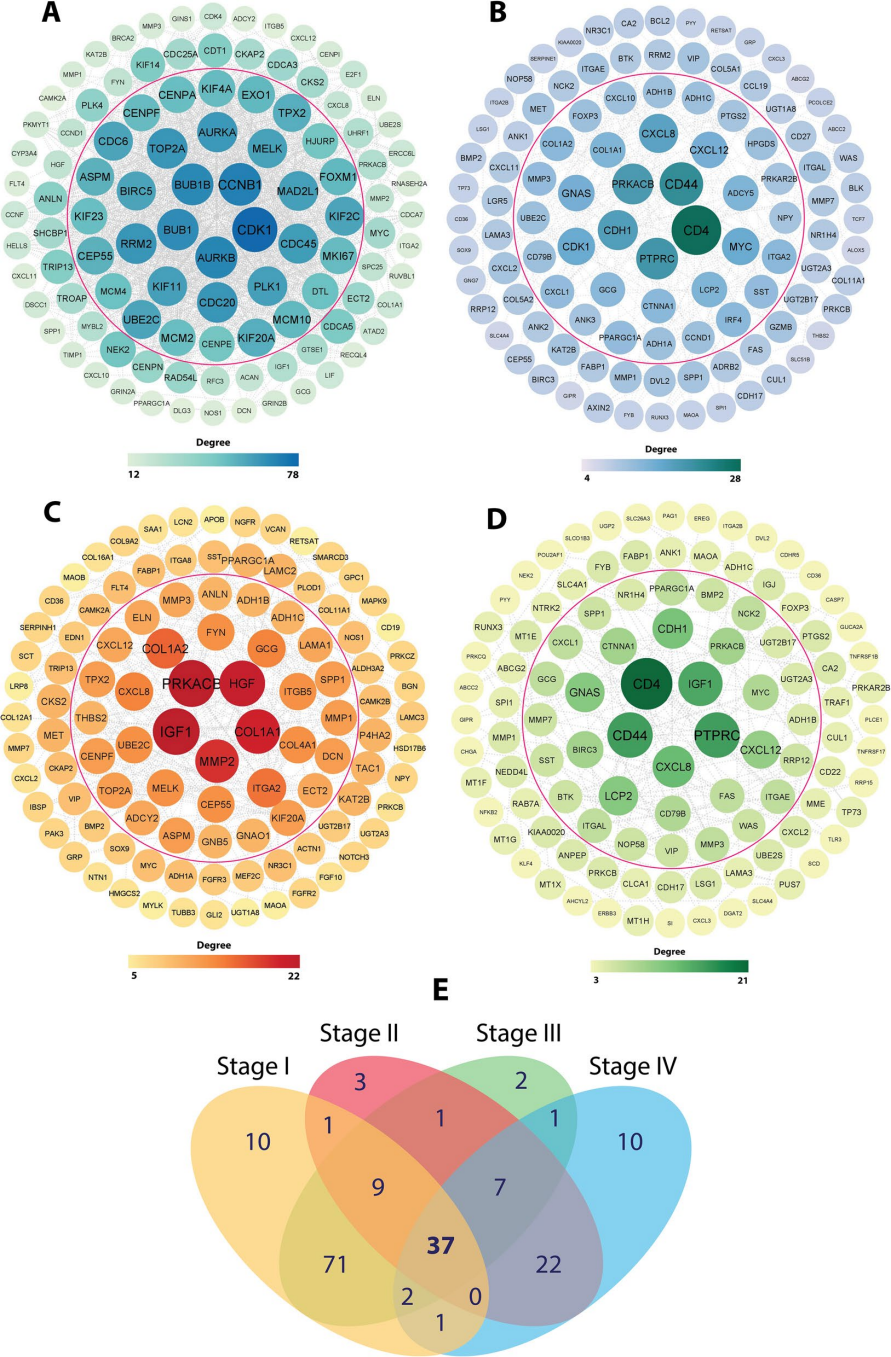
Identification of stage-specific and conserved hub genes. The PPI network of the top genes from the inside to the outside, according to degree from high to low for stage I **(A)**, stage II **(B)**, stage III **(C)** and stage IV **(D)**. Hub genes (top 40 nodes) in PPI network are shown inside the red circle. **(E)** The conserved hub genes, which are common across all CRC stages are selected using VENN diagram

**Fig. 4:**
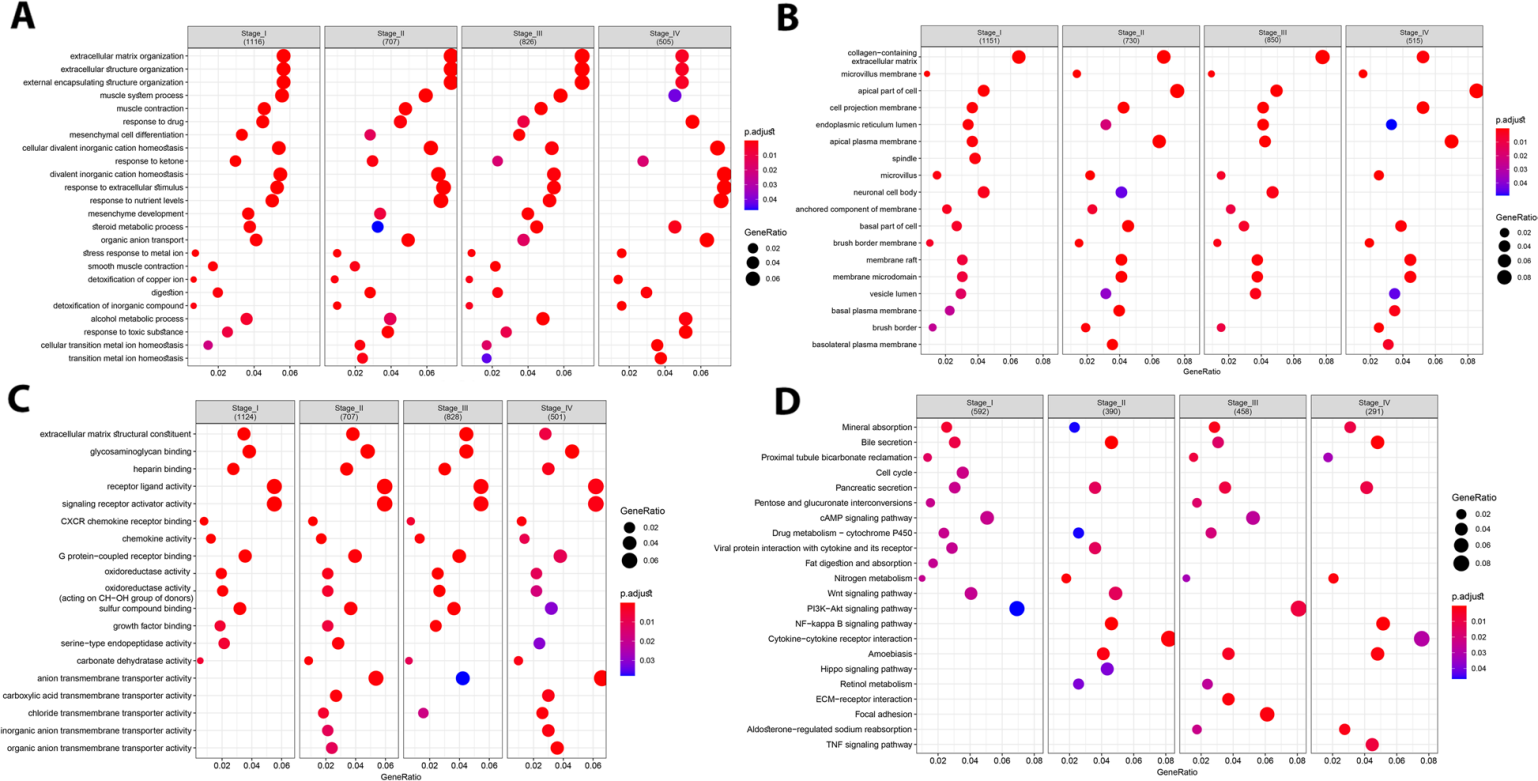
GO and KEGG pathway enrichment analyses of DEGs in each CRC stage using ClusterProfiler. **A** Results of biological process, **(B)** cellular component and **(C)** molecular function as well as **(D)** KEGG pathway enrichment analyses. Size of round node is in proportion to gene ratio of the enriched gene number. GO, Gene Ontology; KEGG, Kyoto Encyclopedia of Genes and Genomes

**Fig. 5:**
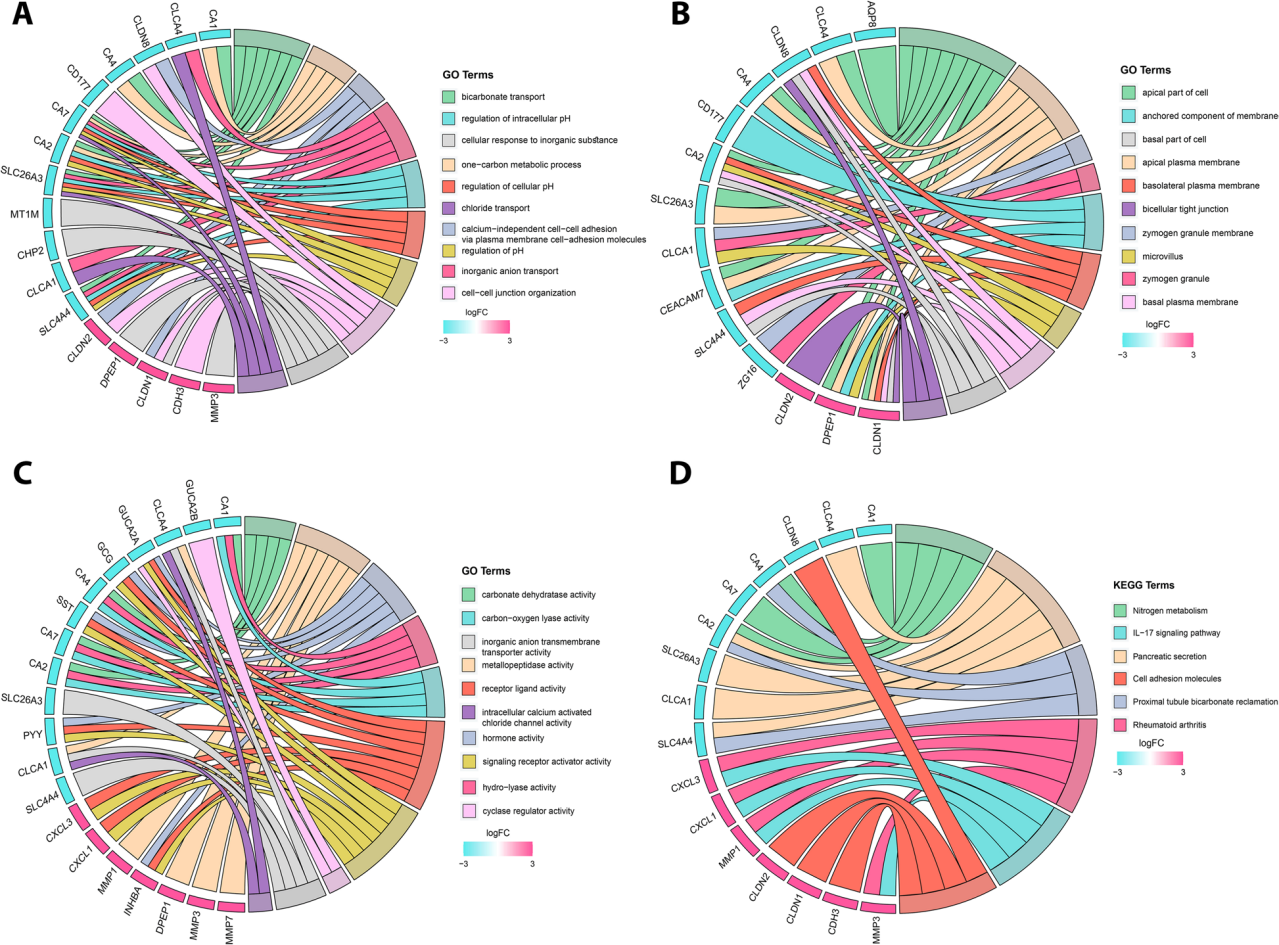
GO and KEGG pathway enrichment analyses of primary hub genes, conserved across all CRC stage using GOplot. **A** Chord plots of biological process, **(B)** cellular component and **(C)** molecular function as well as **(D)** KEGG pathway enrichment analyses (www.kegg.jp/kegg/kegg1.html). Size of round node is in proportion to gene ratio of the enriched gene number. GO, Gene Ontology; KEGG, Kyoto Encyclopedia of Genes and Genomes

In order to determine stage-specific hub genes, we combined DEGs obtained from RRA (Group A) and WGCA (Group B) for stage I (1180 DEGs), stage II (746 DEGs), stage III (897 DEGs), and stage IV (524 DEGs), separately. Then, combined DEGs of stage I to IV were imported into the STRING database to obtain the interaction information (high confidence = 0.7) for the construction of PPI network using Cytoscape software. To determine stage-specific hub genes, network was analyzed and nodes were arranged from the inside to the outside according to the degree to find top 40 UR and DR nodes, depicting the stage-specific hub genes (Fig. 3A-D). Additionally, by setting cutoff criteria for Group A and B (MM > 0.7 in WGCNA, Top 30 UR and DR in RRA), 37 conserved hub genes across all CRC stages were determined to be used for downstream analysis (Additional file 2, Table. S1) (Fig. 3E). For functional enrichment analysis, we have used DAVID to explore the significant terms of GO enrichment (including biological process (BP), cellular component (CC), and molecular function (MF)) and KEGG pathway analyses for both stage-specific and conserved hub genes, separately. Regarding the stage-specific genes (Additional file 2, Table. S2, S3), in the BP category, we identified extracellular matrix organization, extracellular structure organization, external encapsulating structure organization, muscle system process, response to drug, etc. as the most significant processes across all CRC stages (Fig. 4A). In the CC category, collagen-containing extracellular matrix, microvillus membrane, apical part of cell, cell projection membrane, endoplasmic reticulum lumen, apical plasma membrane, etc. were significantly enriched (Fig. 4B). Moreover, extracellular matrix structural constituent, glycosaminoglycan binding, heparin binding, receptor ligand activity, signaling receptor activator activity, CXCR chemokine receptor binding, etc., were among the most significantly enriched GO MF terms (Fig. 4C). Based on KEGG pathway analysis, Mineral absorption, Bile secretion, Proximal tubule bicarbonate reclamation, Pancreatic secretion, etc., were mostly associated with the stage-specific DEGs across all stages (Fig. 4D). For the conserved hub genes, GO and KEGG pathway enrichment analyses were also performed. The top ten enriched terms for 37 genes are shown in Fig. 5A-D. These hub genes are mostly enriched in bicarbonate transport, cell-cell junction, chloride transport, apical part of cell, carbonate dehydratase activity, receptor ligand activity, pancreatic secretion, etc. (Additional file 2, Table. S4). Additionally, we have performed GO and KEGG analyses for DEGs of all CRC stage, which demonstrated enriched pathways as described earlier (Additional file 1. S5).

### Gene expression patterns and chromosome locations of hub genes

For further analysis of hub genes, their Pearson’s correlations and expression patterns across the 12 datasets from Group B, as well as their chromosomal locations were extracted and visualized (Additional file 1. S6A). In this regard, Chromosome 16 contained most hub genes and other DEGs were distributed in all chromosomes except for chromosome 5, 7, 9, 10, 12, 13, 18, 22, and Y. The top 5 UR DEGs according to logFC included MMP7, FOXQ1, MMP3, KRT23, and CDH3, which were distributed in chromosomes 11, 6, 11, 17, and 16. The top 5 DR DEGs were also CLCA4, MS4A12, GUCA2B, AQP8, and CA1, with locations in chromosomes 1, 11, 1, 16, and 8. Moreover, according to PCC calculations, DEGs in the chromosomes 4, 8, 16, and 17 have shown the most significant correlations based on the cut-off criteria of PCC > 0.85 and FDR < 0.05.

### Identification of therapeutic targets based on the mRNA-lncRNA-miRNA and PPI networks

**Fig. 6:**
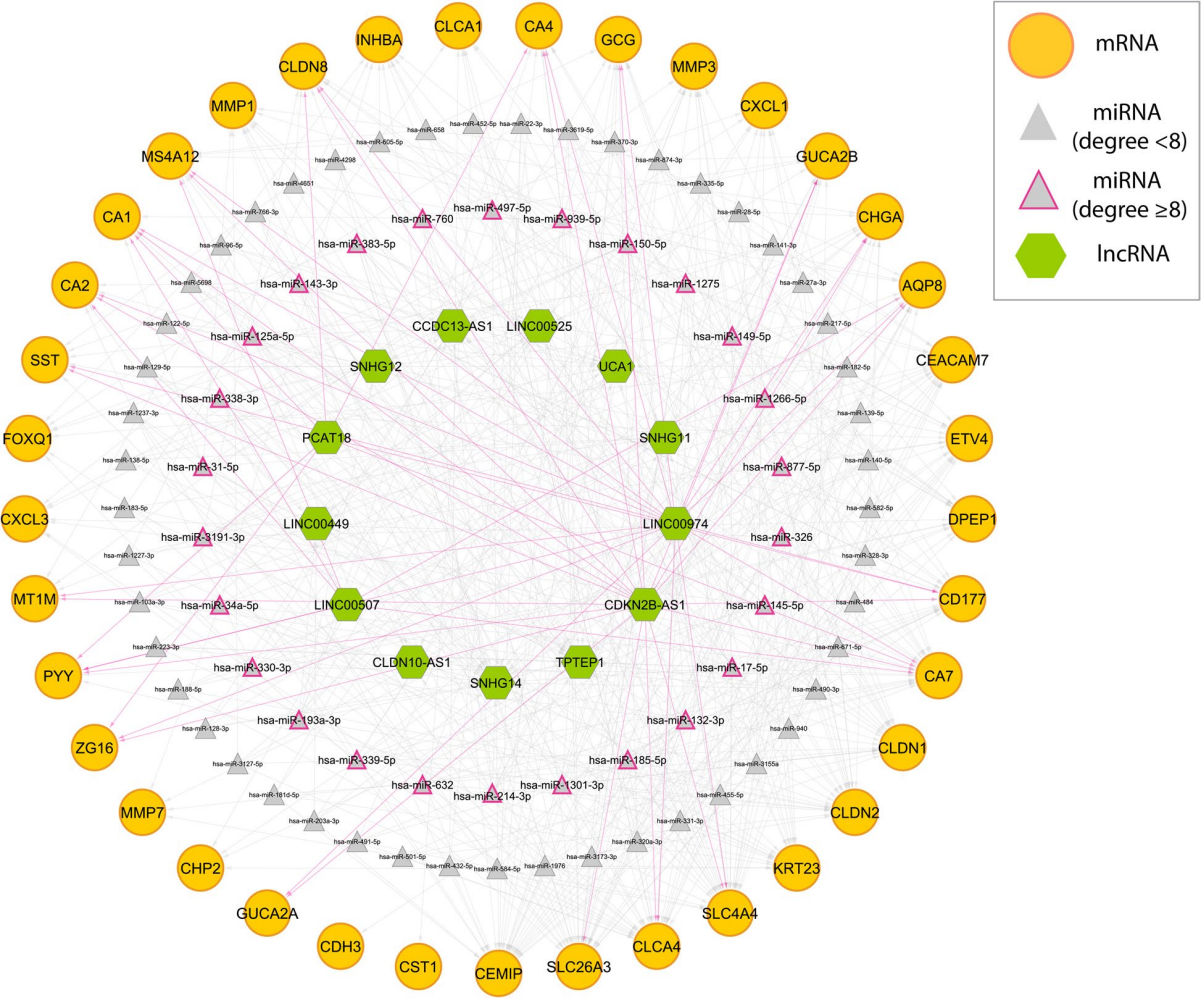
The mRNA-miRNA-lncRNA regulatory network constructed by significantly DEGs, DE miRNAs, and DE-lncRNAs. Different colors indicate different RNA molecules (yellow: mRNAs, gray: miRNAs, and green: lncRNAs). The outer circle includes mRNAs targeted by ncRNAs, the middle circle is miRNAs with the degree less and more than 8, and the inner circle is lncRNAs. Pink and gray lines represented the nodes targeted by lncRNAs and miRNAs, respectively

To construct a mRNA-lncRNA-miRNAnetwork, the significant co-dysregulated competing pairs (containing only DE-miRNAs and DE-lncRNAs) were extracted from each target pairs. In this case, we identified a total of 289 DE-miRNAs and 673 DE-lncRNAs by comparing the CRC with the adjacent normal tissue samples using the edgeR/LIMMA packages (Additional file 3, Table. S1, S2). As a result, there were a total of 752, 195 and 53 reliable miRNA-mRNA, lncRNA-miRNA, and mRNA-lncRNA pairs, respectively, which were used for further analysis (Additional file 3, Table. S3, S4, S5). Given this, we constructed co-regulatory network of different ncRNAs to highlight CRC carcinogenesis based on their multi-reciprocal interactions. By excluding nodes with less than four degrees, the regulatory network was constructed based on potential interactions between target pairs (Additional file 3, Table. S6). Complex reciprocal interaction of different miRNA-mRNA pairs indicated a higher number of edges shared by hsa-miR-1301-3p (11 out of 37), hsa-miR-185-5p (11 out of 37), hsa-miR-326 (10 out of 37), and hsa-miR-145-5p (9 out of 37). Regarding lncRNA-mRNA pairs, CDKN2B-AS1 (19 out of 37), LINC00974 (18 out of 37), PCAT18 (7 out of 37) and LINC00507 (6 out of 37) demonstrated as the key lncRNAs targeting hub DEGs in the proposed regulatory network (Fig. 6). Moreover, SNHG14, TPTEP1, SNHG11, SNHG12, UCA1, LINC00525 have shown potential interaction with 50, 38, 18, 17, 15, and 13 DE-miRNAs, respectively, which may suggest their crucial roles in the CRC carcinogenesis (Fig. 6). Notably, as represented in Fig. 6, SLC4A4, SLC26A3, CEMIP, CLCA4, and CLDN2, represented as the top five important mRNAs in the network, by interacting with 30, 29, 29, 28, and 24 ncRNAs, respectively.

The MODE plugin in Cytoscape was also used to identify the potential core hub genes in the network. In this regard, DEGs with the highest MCODE score of 7.7 were all DR and included CLCA1, AQP8, ZG16, GUCA2A, CLCA4, GUCA2B, SLC26A3, MS4A12 according to the degree from 6 to 10. These hub genes along with the core miRNAs and lncRNAs mentioned above are considered as the most important therapeutic targets across all CRC stages (Additional file 1. S6B). However, core mRNA biomarkers listed above has been reported recently in literature ([31–37]), and they mainly regulated by the function of more important ncRNAs. Moreover, lncRNAs comprise a large proportion of the transcriptome and many recent studies have shown their important roles in the initiation and progression of cancer via different mechanisms. These are included epigenetic gene regulation, gene splicing, mRNA stability and protein translation, as well as acting as decoys or “sponges” for miRNAs or transcription factors. On the other hand, the fact that lncRNAs are mainly expressed in a cell- or tissue/tumor specific manner makes them exceptional therapeutic targets. Given this, we have focused on lncRNAs with higher degree of connectivity among the nodes rather than mRNAs or miRNAs, based on their importance, novelty as well as the ability to directly target hub genes (DEGs) in the regulatory network of CRC carcinogenesis. Therefore, CDKN2B-AS1, LINC00974, PCAT18 and LINC00507 were chosen for further assessments.

### Expression and immunohistochemistry validation of therapeutic targets in silico

**Fig. 7:**
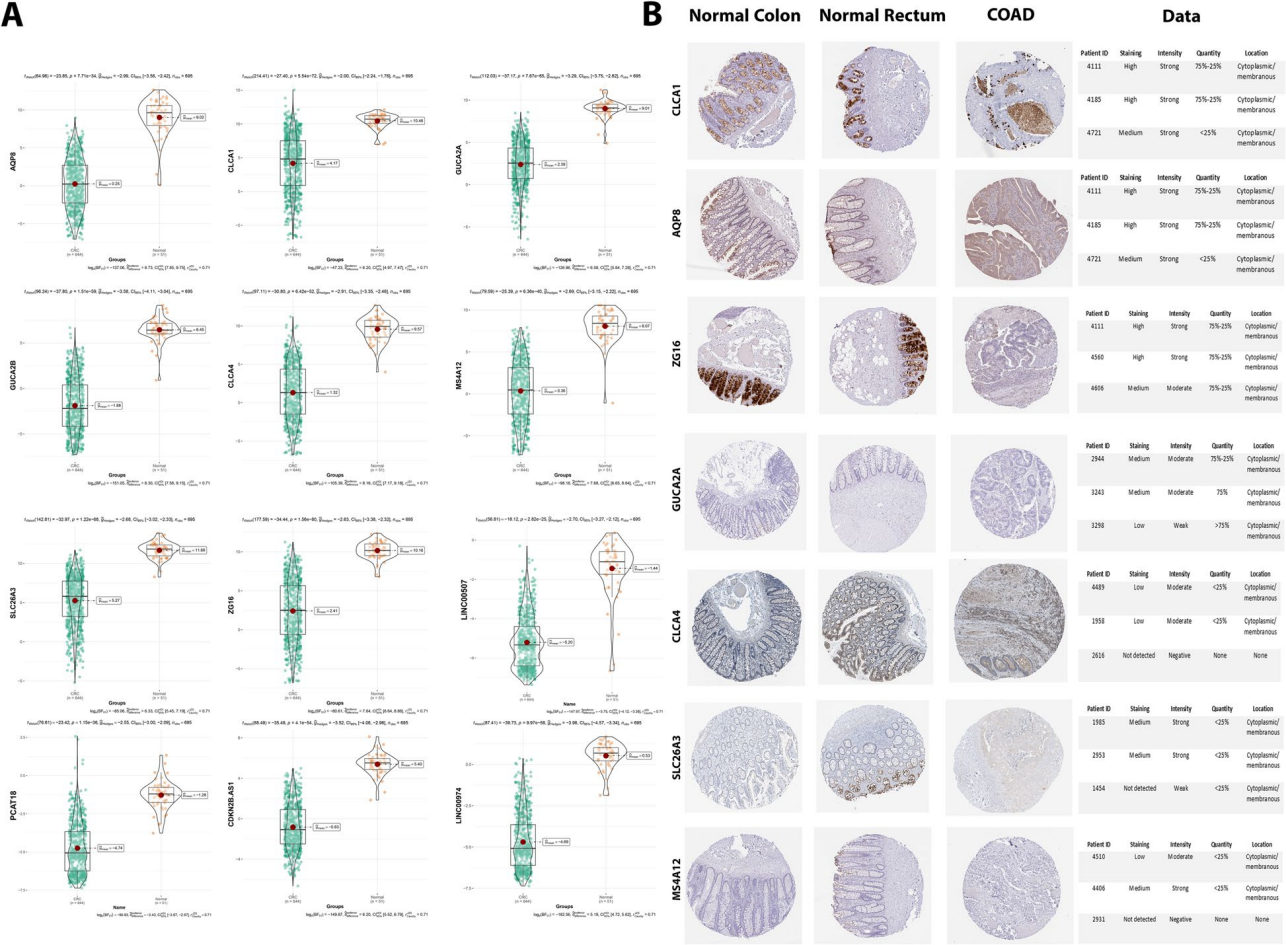
Validation of final eight hub genes (mRNAs) in the TCGA COAD-READ database. **A** Expression of CLCA1, AQP8, ZG16, GUCA2A, CLCA4, GUCA2B, SLC26A3, and MS4A12 as well as CDKN2B-AS1, LINC00974, PCAT18 and LINC00507 in CRC samples versus normal samples and in **(B)** Validation of protein expression for CLCA1, AQP8, ZG16, GUCA2A, CLCA4, SLC26A3, and MS4A12 in CRC tissues compared to normal COAD and normal READ

The expression analysis of final eight hub genes as well as final four lncRNAs using TCGA COAD-READ dataset represented significantly lower expression in CRC tissues compared with normal tissues (Fig. 7A). The pattern of expression did not vary between different CRC stages, which indicated their conserved expression (Additional file 1. S7). Moreover, protein expressions of the eight hub genes were validated using immunohistochemistry images from the Human Protein Atlas database (Fig. 7B). In this regard, the protein expression of seven hub genes (except GUCA2B, with no IHC information) were significantly higher in normal colon and rectum tissues than that in COAD tissue, which validated previous results from expression analysis.

### Identification of potential biological functions of hub genes

**Fig. 8:**
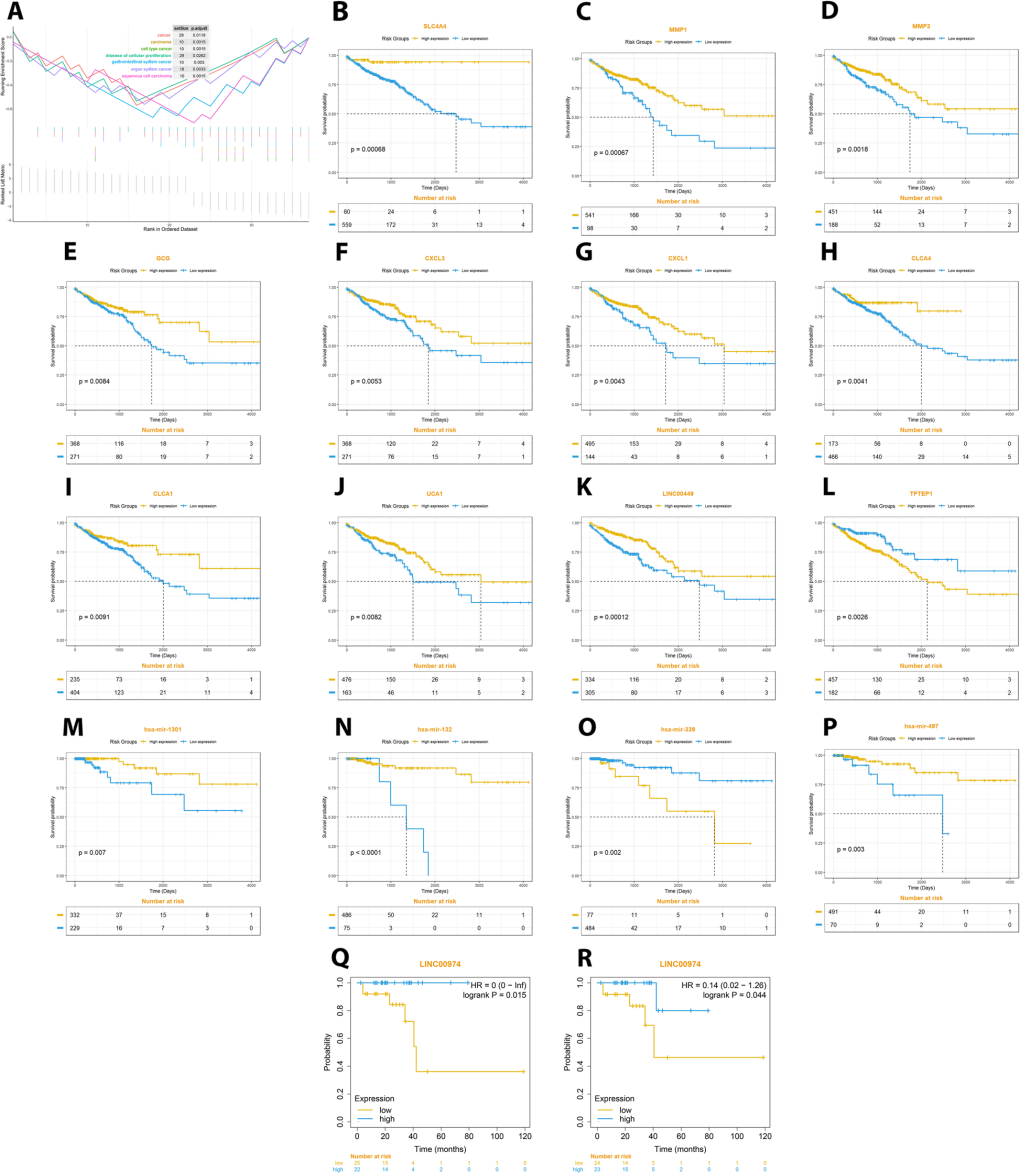
**(A)** Gene set enrichment analysis (GSEA) of hub genes in the TCGA COAD-READ dataset. The association between expression of **(B)** SLC4A4, **(C)** MMP1, **(D)** MMP3, **(E)** GCG, **(F)** CXCL3, **(G)** CXCL1, **(H)** CLCA4, and **(I)** CLCA4 as mRNAs, besides **(J)** UCA1, **(K)** LINC00449, and **(L)** TPTEP1 as lncRNAs, and **(M)** has-miR-1301, **(N)** has-miR-132, **(O)** has-miR-339, and **(P)** has-miR-497 as miRNAs with OS of all patients in the TCGA COAD-READ dataset. Moreover, Kaplan-Meier analyses of DFS for LINC00974 with the **(Q)** optimal**-** and **(R)** median-cut-off values in patients with CRC have been shown. The yellow line indicates high expression groups and the blue line represents the low expression group

To explore the biological functions of hub genes, GSEA method was used based on TCGA COAD-READ dataset. As represented in (Fig. 8A), our primary hub genes were enriched in different pathways of disease of cellular proliferation (for 20 hub gene out of 37), cancer (20/37), organ system cancer (15/37), carcinoma, cell type cancer, gastrointestinal system cancer and squamous cell carcinoma (9/37) with an FDR of < 0.05 (Additional file 4, Table. S1). Moreover, the enriched functional terms of CDKN2B-AS1, LINC00974, PCAT18 and LINC00507 were also the same as these lncRNAs have interactions with many of hub genes out of 37. These findings showed biological significance of this four lncRNAs in CRC initiation and progression.

### Identification of biomarkers with potential prognostic value

The Kaplan–Meier survival analysis stratified by the optimal cutoff value showed 8 mRNAs, 3 lncRNAs and 4 miRNAs that were significantly related to the poor OS of CRC patients (Fig. 8B to 8P, p < 0.01). In this regard, MMP1 (*p =* 0.00067), SLC4A4 (*p =* 0.00068), LINC00449 (*p =* 0.00012) and has-miR-132 (*p* < 0.0001) predicted the most significant poor OS in CRC patients. Moreover, out of 37 primary hub genes (mRNAs), 14 of them were also shown to be significantly correlated with poor OS (*p* < 0.05). However, the clinical significance of four-lncRNA signature, was not related to the poor OS and we further evaluated DFS among CRC patients using online tool KM plotter. In this case, only LINC00974 was significantly related to DFS of CRC patients, with *p =* 0.015 and *p =* 0.044 for optimal and median cut-off values, respectively (Fig. 8Q, R). The obtained results confirmed that the protein expression of primary hub genes as well as several hub lncRNAs and miRNAs are related to the progression and worse clinical outcomes of CRC patients.

### Identification of biomarkers with potential diagnostic value

**Fig. 9:**
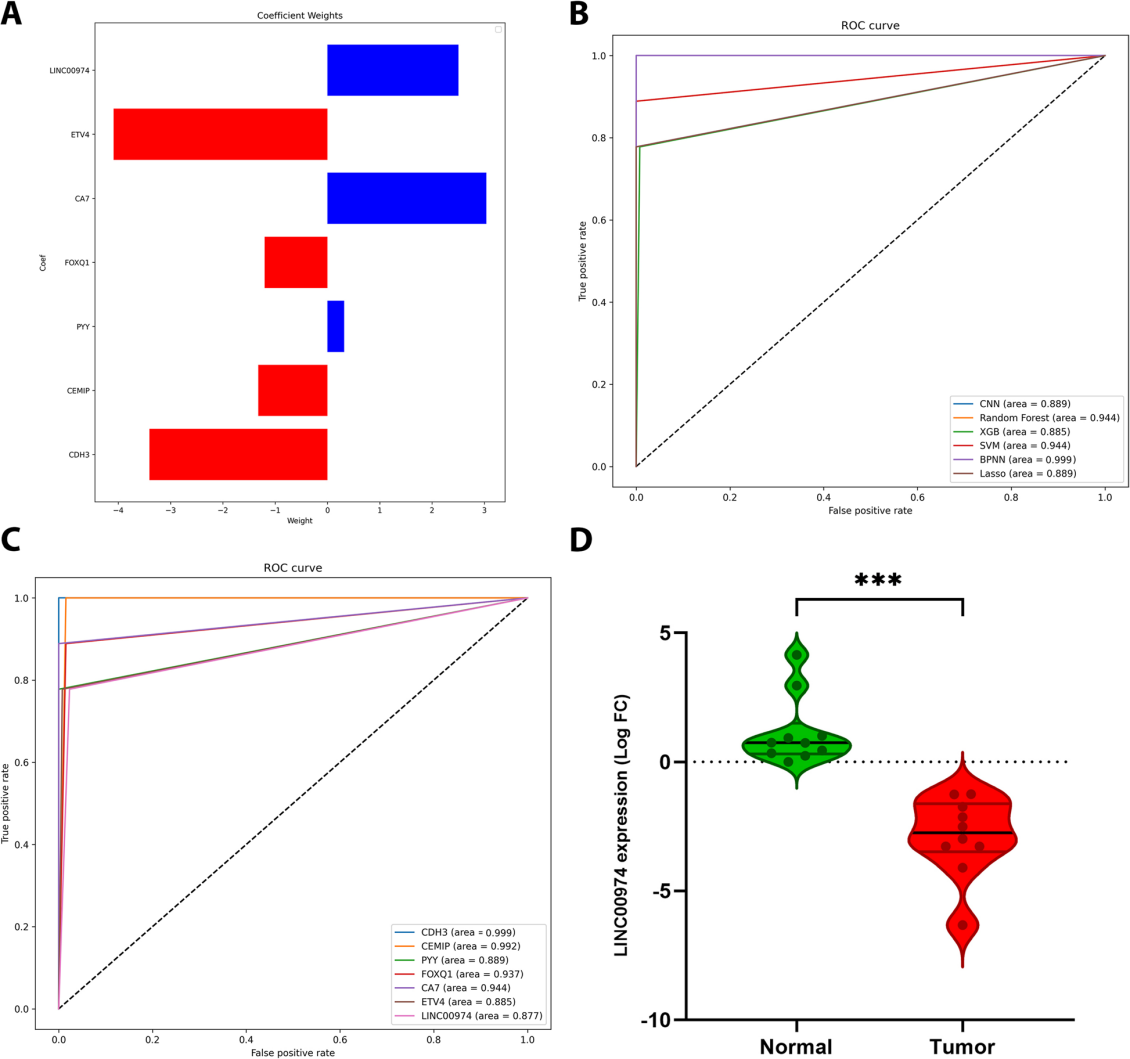
**(A)** Feature selection using LASSO. where the non-zero coefficients of 7 mRNA and lncRNA as features was calculated and displayed. **B** The ROC curve showed the prediction accuracy of different six models on the test set. The AUC value of each model represents the area under the ROC curve. The closer the AUC to 1, the better the generalization of the model. **C** The back-propagation neural network (BPNN) provided the most efficient estimates. **D** RT-qPCR analysis of LINC00974 expression in 10 CRC and adjacent normal tissue samples

The ML and DL approaches in our study were based on pre-defined lists of hub genes (mRNAs and lnRNAs) and miRNAs with their expression levels from TCGA COAD-READ. Firstly, LASSO algorithm was employed for selection of appropriate hub genes for downstream ML and DL model validation. In this case, the λ value was defined as the penalty coefficient, by which for hub genes 7 features were found based on their weights for the construction of the predictive model (Fig. 9A). Following this step, we evaluated the performance of six different ML and DL models (LASSO, XGBoost, RF, and SVM as ML algorithms, besides BPNN and CNN as DL approaches) using ROC and measuring AUC value that plots TPR against FPR at various threshold values (Fig. 9B, C). Therefore, the model with a higher AUC value was selected to build a more robust prediction. In this regard, a simple comparison of six ML and DL models based on the AUC, displayed BPNN (AUC = 0.999) for performing the best model validation for both mRNAs and lncRNAs among other models; while RF and SVM and RF have also shown an appropriate AUC for model prediction. As shown in Fig. 9C, after setting iterations to 1000 to train BPNN model, the fitting effect enhanced with the number of iterations, which was resulted in lower loss value. As a result, based on the AUC values, CDH3 as mRNA (Fig. 9C) and has-miR-3173 and has-miR-141 from miRNAs (Additional file 1. S8, A:C) have represented the highest diagnostic power in distinguishing between CRC from non-cancerous tissues (AUC = 0.999). Furthermore, CEMIP, CA7 and FOXQ1 with AUC values of 0.992, 0.944, and 0.937, have shown good diagnostic values, respectively (Fig. 9C). Regarding lncRNAs, only LINC00974 showed a good diagnostic efficacy with an AUC value of 0.87, which suggested this lncRNA as a promising diagnostic and prognostic tissue biomarker as well as potential therapeutic target for CRC (Fig. 9C). Therefore, we have chosen LINC00974 for further assessments in CRC tissues through RT-PCR.

### LINC00974 is significantly downregulated in CRC tissues

Since lncRNA LINC00974 has been represented as a potential and novel therapeutic target with a high diagnostic power in CRC, its expression level was investigated in CRC (*n =* 10) and adjacent normal tissues (*n =* 10), using RT-qPCR. As shown in Fig. 9D, the expression of LINC00974 in CRC tissues was significantly lower than that in adjacent normal tissues (*p =* 2e− 04). In addition, an external validation using GSE87211 samples have demonstrated significant under-expression of LINC00974 in CRC versus normal samples (Additional file 1. S8, D). Therefore, in similar to TCGA expression analysis, LINC00974 was also downregulated in CRC compare to normal tissues.

## Discussion

CRC is the most commonly diagnosed gastrointestinal cancer, and the current therapeutic strategies as well as its diagnosis and prognosis have limitations [38]. Therefore, identifying potential therapeutic targets at the molecular level, and biomarkers with robust diagnostic and prognostic value for CRC based on a stage-specific strategy seems necessary [39]. In the current study, for the first time we aimed to combine RRA analysis with WGCNA to explore the significant genes associated with different stages of CRC (stage I to stage IV). To explore potential DEGs between CRC tumor tissues and normal tissues we integrated 21 GEO and RNA-seq datasets to provide a robust list for subsequent analysis of stage-specific and conserved hub genes. In this regard, the results indicated that CDK1 and CCNB1 are the top two core genes in the early stages of CRC, which have been shown by J Li et al .[40] and Y Fang et al .[41], respectively. Regarding stages II to IV of CRC, the key genes were CD4, CD44, PRKACB and IGF1, which in different studies their alterations implicated in the CRC. For instance, in a study by Xia et al. they have displayed that CD44 mRNA level was correlated with a benign survival rate in colon and gastric cancers, and it may be used as biomarkers to predict the prognosis of colon cancer patients [42]. Interestingly, Yao et al. in their work observed that PRKACB is down regulated in patients with stage III-IV colorectal cancer (HR = 2.931 (1.357-6.333), log-rank *P =* 0.0145), but not identified as risk factors for patients with stage I-II colorectal cancer (*P >* 0.05). So, this down-regulated expression of PRKACB in tumors associated with worsening OS in CRC patients [43]. Furthermore, in another work conducted by Qiao et al. it has been represented that IGF1-mediated HOXA13 overexpression was associated with histological grade, T stage, N stage, M stage (stage II to IV), and tumor size in CRC tissues. Therefore, by targeting the IGF1-HOXA13-IGF1R oncogenic loop, a potential therapeutic strategy could be introduced to inhibit HOXA13-driven CRC metastasis [44].

We have also used GO and KEGG enrichment analyses to explore the functions of the stage-specific DEGs identified by combining the RRA and WGCNA methods. GO analysis indicated that dysregulated extracellular matrix and structure as well as alterations in collagen containing extracellular matrix, cell projection membrane, receptor ligand activity, etc. were significantly related to the development and progression of cancer from stage I to IV [45]. Additionally, in KEGG pathways, cell cycle only enriched in DEGs from stage I of CRC, and pathways like bile secretion and nitrogen metabolism were enriched in all stages of CRC, which their potential mechanisms in CRC risk and pathogenesis were indicated in several studies [46]. While, nitrogen is a vital biomolecule in human metabolisms and regulates cellular metabolism and a shift in glutamine nitrogen metabolism may thus be required for malignant progression of colorectal cancer [47].

In the second part of this paper, we have analyzed conserved hub genes and the core genes within these hub genes (extracted by MCODE) through several approaches to address their potential therapeutic, diagnostic and prognostic value for CRC patients. Based on the results of the GO analysis, DR hub genes were mainly enriched in hypoxia related pathways of bicarbonate transport, regulation of cellular and intracellular pH [48], while, cell-cell junction organization and calcium-independent cell-cell adhesion were mainly identified for UR genes from the category of BP. Moreover, for CC, hub genes were mainly identified for apical part of the cell and apical plasma membrane, which their dysregulation promotes cell transformation, migration and metastasis outgrowth [49]. MF and KEGG analyses also showed enrichment in multiple tumor-related pathways including receptor ligand activity, metallopeptidase activity, nitrogen metabolism, pancreatic secretion, etc., which are all implicated in the tumor cell proliferation and the progression of CRC.

Regarding final eight hub genes, CLCA1 and CLCA4 as chloride channel accessories were identified in three GO categories as well as KEGG pathways, and they both have shown crucial roles in the pathogenesis of CRC. In this regard, in a study conducted by Li et al., they investigated the molecular functions and mechanisms of CLCA1, by constructing CLCA1-upregulated cells and CLCA1-knockout cells in the SW620 cell line of CRC. In vitro and in vivo results of this study suggested that CLCA1 plays a key role as a tumor suppressor in CRC by suppressing the epithelial–mesenchymal transition (EMT) and Wnt/beta-catenin signaling pathway [31]. Besides CLCA1, it has also been showed that CLCA4 contributes to the progression of several types of malignant tumors including CRC. In this respect, Chen et al. have provided the evidence that cell models with CLCA4 gain-of-function, migration and invasion of SW620 and LoVo cell line inhibited through suppressing EMT via PI3K/ATK signaling in vitro. The results of this study implicated that CLCA4 may help to discriminate potential risk of lymph node metastasis in CRC patients [50]. SLC26A3 as an intestinal Cl−/HCO3- exchanger, which is enriched in several terms and present in the apical domain of multiple intestinal segments have also shown significantly downregulation in CRC patients. In this relation, in a few numbers of studies it has been proposed that SLC26A3 primarily expressed in colon cancer, but its expression level is significantly decreased in CRC, indicating its association with the progression of CRC. Thus, downregulated SLC26A3 may be considered as a marker of differentiated colonic epithelial cells rather than a marker of cell proliferation [51, 52]. Low levels of AQP8, which only enriched in apical part of the cell, have revealed to significantly promotes cancer growth and metastasis. While, its over-expression inhibited CRC cells growth, mobility and aggressiveness by inactivating PI3K/AKT signaling and inhibiting PCDH7 expression in vitro and in vivo [32]. Another hub gene which is only enriched in zymogen granule, is ZG16, as one of the most significantly downregulated genes in CRC [33]. In another study it has also been demonstrated that ZG16 may keep bacteria further away from the host colon epithelium, indicating an important role of ZG16 in colon surveillance system [53]. Moreover, GUCA2A and GUCA2 were shown to be significantly enriched in cyclase regulator activity and receptor ligand activity. Loss of these hub genes, which is fairly common in intestinal diseases, also rarely evidenced to be linked with CRC pathogenesis and disruption of intestinal homeostasis. In this respect, in a study by Bashir et al., they have investigated the silencing effect of GUCA2A-GUCY2C signaling axis in the tumorigenesis of CRC. As a result, tumor progression through the APC pathway involves loss of the hormone GUCA2A that suppresses the tumor-suppressing receptor GUCY2C. So, hormone replacement (GUCA2A and GUCA2B) restores GUCY2C signaling to prevent MSI tumors, which, makes it to be considered as a novel therapeutic paradigm to prevent CRC tumorigenesis [34]. Lastly, MS4A12 showed no significant enrichment in GO and KEGG terms, however, it participates in different processes of cellular differentiation and proliferation, cell membrane composition, and also cell cycle regulation [54]. However, among rare studies, one study conducted by HE et al. confirming the key role of MS4A12 during CRC tumorigenesis processes. In this case, their findings were confirmed that after silencing MS4A12, the CRC cell line LoVo cells showed significant resistance to sodium butyrate function of inducing cell cycle arrest and apoptosis, which indicated MS4A12 to be related with cell differentiation of CRC [37]. Taken together, although molecular mechanisms of all eight hub genes mentioned above are still needed to be elucidated, but they might be considered as potential therapeutic targets for future studies in CRC.

Another part of this study was based on complex network-based investigation of crucial mRNAs, miRNAs and lncRNAs, strongly interacted with each other in CRC. The miRNAs that were significantly correlated with primary hub genes (37 DEGs) and rarely reported as therapeutic targets were hsa-miR-1301-3p [55], hsa-miR-185-5p [56], hsa-miR-326 [57], and hsa-miR-145-5p [58]. However, these miRNAs were evaluated for specific signaling pathways, and our results have shown more key targets from our primary list of considered hub genes in CRC. In the present study, we have also introduced rarely reported CDKN2B-AS1, LINC00974, PCAT18 and LINC00507 as core lnRNAs, with the ability to target many hub genes, directly. Interestingly, in two very recent studies, CDKN2B-AS1 have been investigated as a tumor oncogene (upregulated) which regulates cell proliferation and inhibit apoptosis in CRC through sponging miR-28-5p and targeting MAPK inactivator dual-specificity phosphatase 1, respectively [59, 60]. Furthermore, lncRNA PCAT18 have found as another core lncRNA (with less targets), which evidenced to promote CRC tumorigenesis by binding to miR-759 [61]. However, regarding LINC00974 and LINC00507, there are no studies reported their biological importance in CRC and future studies are seemed necessary.

In the current study, in addition to identifying various therapeutic target based on regulatory network, we also evaluated potential prognostic and diagnostic biomarkers via survival analysis and different algorithms of AI (ML and DL) in CRC, respectively. In this respect, eight mRNAs (SLC4A4, MMP1, MMP3, GCG, CXCL1, CXCL3, CLCA1, and CLCA4), three lncRNAs (UCA1, LINC00449, TPT3P1) and four miRNAs (has-miR-1301, has-miR-132, has-miR-339, has-miR-497) were found to be significantly (*p* < 0.01) related with poor OS of CRC patients. A majority of these hub genes were recently investigated as potential prognostic biomarkers, bioinformatically and experimentally [62–65]. Moreover, in our multivariate Cox model, our results suggested that out of four final lncRNAs, LINC00974 was the only lncRNA shown as a novel and potential predictor of DFS in patients with CRC, which is also reported by Gao et al. in gastric carcinoma [66].

A relatively limited number of studies were employed the potential of AI to evaluate the biological significance of genes in cancer. In this study, core biomarkers (mRNAs, miRNAs and lncRNAs) selected by LASSO, were employed for validation by different ML and DL models. In this case, the results represented that AUC of algorithms (RF, SVM and BPNN) exceeded 0.9, especially in BPNN (with an AUC of 0.999), which was higher than the AUC obtained by the previous reports [67, 68]. Then, the BPNN-based model validation for seven hub genes and five miRNAs, showed CDH3, has-miR-3173 and has-miR-141 (with the highest AUC values) as potential diagnostic biomarkers. Interestingly, in very recent studies, CDH3 (also known as Cadherin-3 or Placental-Cadherin), have been shown to be served as an accurate survival predictor in lung cancer [69] and poor prognosis biomarker among patients with tongue squamous cell carcinoma [70], indicating its importance in other cancers. However, there is no current experimental data confirming its diagnostic value for CRC patients, except for a bioinformatic analysis, suggesting its good prognosis for colon adenocarcinoma patients [71]. Additionally, in several studies has-miR-141 (or microRNA-141) have shown as an oncogene, which its suppression inhibits proliferation and migration of CRC cells [72]. So, its differentially expressed serum levels may be used as novel biomarkers for early detection of liver metastasis in CRC [73]. Concerning miR-3173, however, there is no report investigating its roles in CRC tumorigenesis, while it has been introduced as a prognostic marker of poor survival in ovarian carcinoma [74]. Based on the AUC, LINC00974 had a good diagnostic power in distinguishing between cancerous and non-cancerous tissues among lncRNAs. On the other hand, experimental validation of this lncRNA, represented significant downregulation in CRC tissues rather than adjacent normal tissues, confirming its potential as a therapeutic target.

Although the functional role of LINC00974 in the progression of CRC has not been reported yet, but the findings from several recent studies showed its key roles in the progression of different cancer types [75–78]. In this case, in a very recent study, Liu et al. investigated functional roles of LINC00974 in the ovarian cancer development by sponging miR-33a. As a result, they have shown upregulation of LINC00974 and HMGB2 mRNA expressions in ovarian cancer cells, while miR-33a expression (which directly targets HMGB2) was downregulated. So, silencing of LINC00974 by sponging miR-33a suppressed cell proliferation, invasion and EMT of cancer cells, suggesting the importance of LINC00974/miR-33a/HMGB2 axis in the progression of ovarian cancer [77]. In another study conducted by Tang et al. they explored the regulatory mechanisms of LINC00974 and KRT19 in hepatocellular carcinoma. In this case, knockdown of LINC00974 resulted in an inhibition of cellular proliferation and invasion with induction of apoptosis and cell cycle arrest in vitro, which was also validated by xenotransplantation model in vivo. They also discovered that increased level of LINC00974 is a result of abnormal promoter hypomethylation, which induced the upregulation of KRT19 via ceRNA interaction, resulting in the activation of the Notch and TGF-β pathways. Therefore, our data suggested that lncRNA LINC00974 downregulation was significantly associated with aggressive progression and poor prognosis in CRC patients. LncRNA LINC00974 was identified for the first time as potential biomarker for targeted therapy, predicting the clinical outcome and diagnosis of CRC patients. However, further studies are needed to elucidate the mechanisms of action of LINC00974 lncRNA in CRC [78]. Regarding the limitations in the present study, we can address the following items: first, in the retrospective studies (such as microarray and RNA-seq datasets) these were heterogeneity in the results; thus, more in vivo and in vitro experiments should be performed to validate our findings. Second, for evaluating the biological significance of potential biomarkers identified in this study, further investigations are warranted.

## Conclusions

In conclusion, we identified stage-specific and conserved DEGs that were found to be dysregulated in the progression of CRC using WGCNA and RRA analysis. Then, by constructing regulatory network we revealed the most important therapeutic targets. Moreover, we employed several downstream analyses such as survival analysis and six different machine learning and deep learning algorithms to identify key biomarkers with the highest ability for prognosis and diagnosis in CRC. Finally, these findings beside expression validation by RT-qPCR, revealed LINC00974 as a novel index for clinical prognosis and diagnosis of tumor growth and metastasis, which can be evaluated as a potential therapeutic target in future studies of CRC targeted therapy.

## Supplementary Information

**Additional file 1.**

**Additional file 2.**

**Additional file 3.**

**Additional file 4.**

## Data Availability

The datasets generated and/or analyzed during the current study are available in the [https://www.ncbi.nlm.nih.gov/geo/database] and https://portal.gdc.cancer.gov/]. The original contributions presented in the study are included in the article/Supplementary Material, further inquiries are available from the corresponding author on reasonable request.

## Abbreviations

CRC: Colorectal cancer
DEG: Differentially expressed gene
RRA: Robust Rank Aggregation
WGCNA: Weighted gene co-expression network analysis
LncRNAs: Long non-coding RNAs
mRNA: Messenger RNA
ML: Machine learning
DL: Deep learning
AI: Artificial intelligence
PPI: Protein–protein interaction
COAD: Colon adenocarcinoma
READ: Rectal adenocarcinoma
PCA: Principal component analysis
GO: Gene ontology
KEGG: Kyoto Encyclopedia of Genes and Genomes
GSEA: Gene Set Enrichment Analysis
LASSO: Least absolute shrinkage and selection operator
SVM: Support vector machine
RF: Random Forest
Xgboost: eXtreme Gradient Boosting
BPNN: Back propagation neural network
CNN: Convolutional neural network
ROC: Receiver operating characteristic
AUC: Area under curve
