# Supplementary material for: An integrative transcriptome analysis reveals potential predictive, prognostic biomarkers and therapeutic targets in colorectal cancer
Source: BMC Cancer. 2022 Jul 30;22:835. doi: 10.1186/s12885-022-09931-4 (PMC9339198; doi:10.1186/s12885-022-09931-4)
Supplement: Supplementary file 1 — Additional file 1. [file 12885_2022_9931_MOESM1_ESM.docx]

**An Integrative Transcriptome Analysis Reveals Long Noncoding RNA LINC00974 as a Potential Therapeutic Target in Colorectal Cancer**

**Pouria Samadi, Meysam Soleimani**^2^**,** **Fatemeh Nouri**^2^**, Fatemeh Rahbarizadeh**^3^**, Rezvan Najafi**1**, Akram Jalali^*^**

Research Center for Molecular Medicine, Hamadan University of Medical Sciences, Hamadan, Iran

^2^ Department of Pharmaceutical Biotechnology, School of Pharmacy, Hamadan University of Medical Sciences, Hamadan, Iran

^3^ Department of Medical Biotechnology, Faculty of Medical Sciences, Tarbiat Modares University, Tehran, Iran

^*^ **Correspondence**:

**Akram Jalali**, Research Center for Molecular Medicine, Hamadan University of Medical Sciences, Hamadan, Iran, akram.jalali59@gmail.com

**Supplementary Figures:**

**
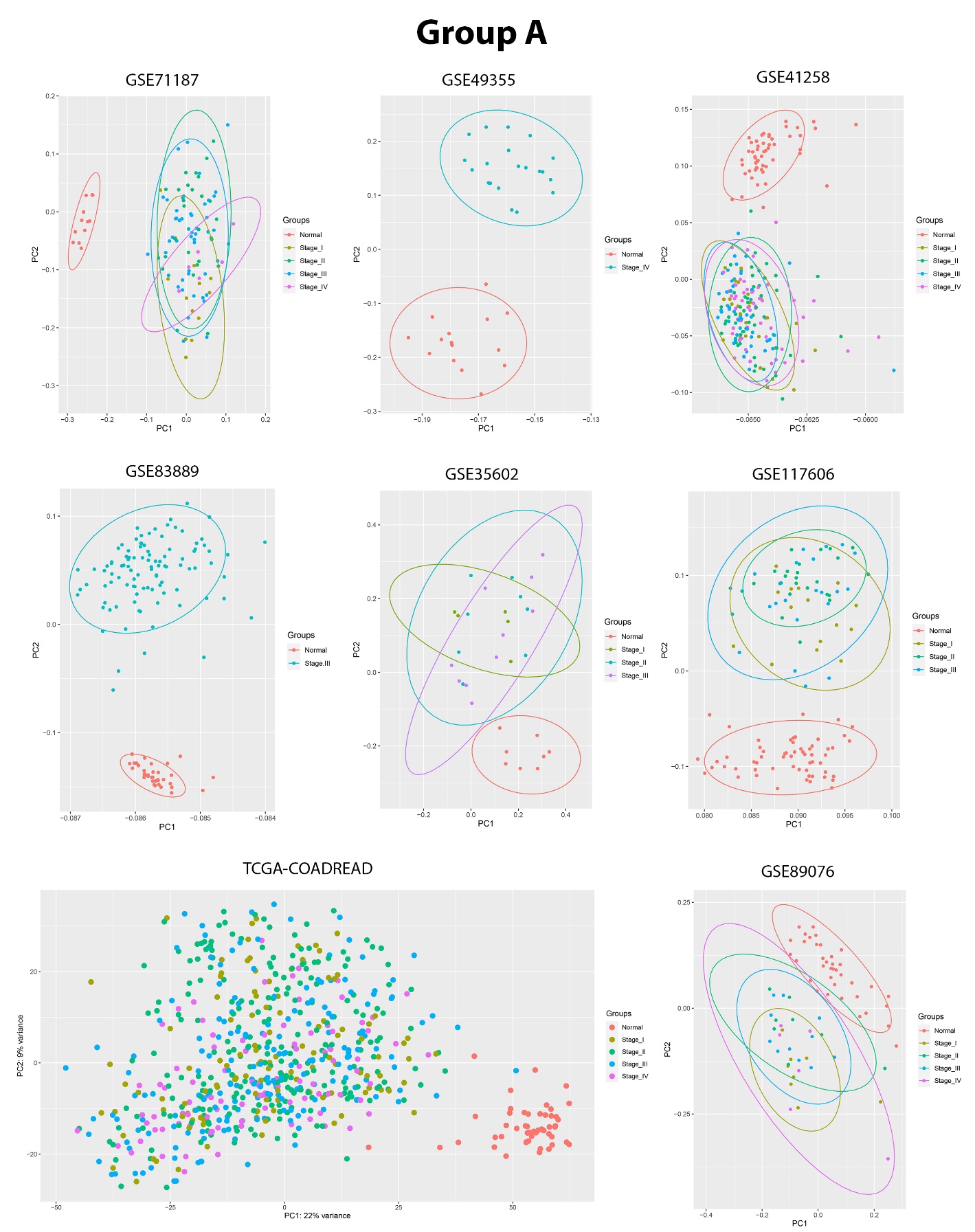
**

**Supplementary Figure S1:** PCA plots of all the datasets in Group A, after removing outlier samples.

**
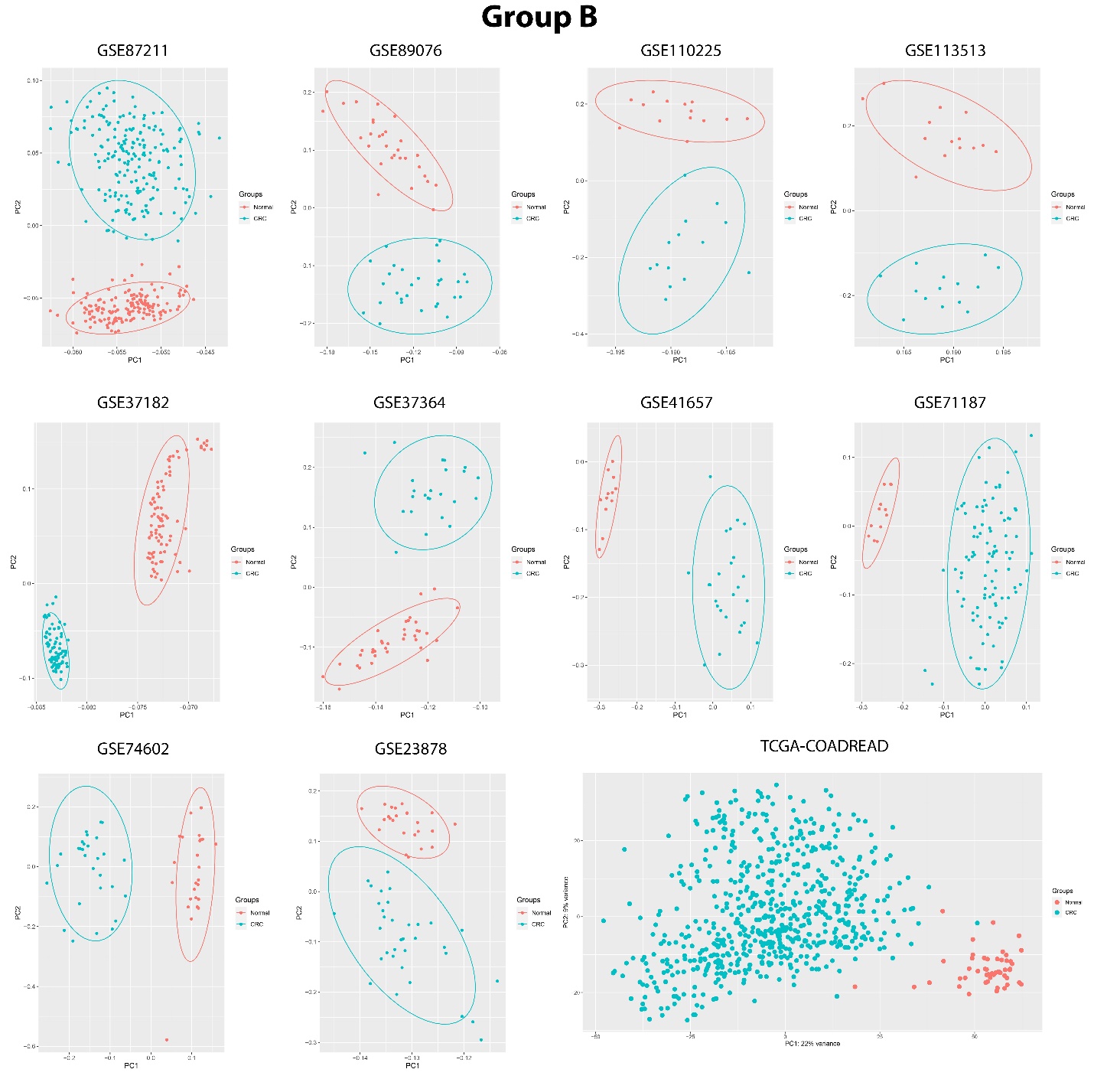
**

**Supplementary Figure. S2:** PCA plots of all the datasets in Group B, after removing outlier samples.


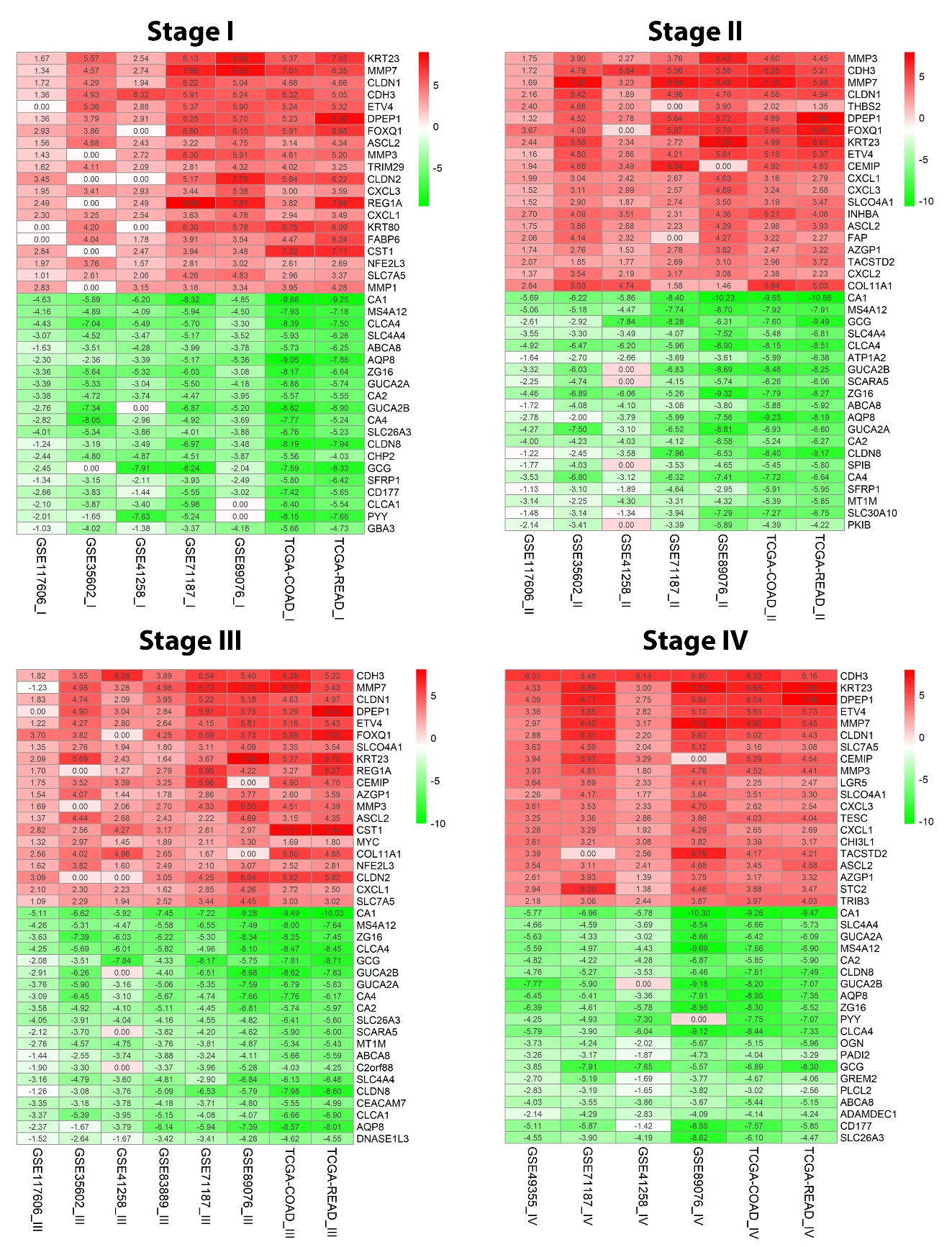


**Supplementary Figure. S3:** Based on the logarithmic fold change value of each DEG, the top 20 up and down regulated robust DEGs analyzed in Group A (stage I to IV) by RRA are shown in this heatmap. The value of “0” indicates that the gene corresponding to the row is missing in the data set corresponding to the column. Red cells indicates that DEGs are upregulated in CRC samples, while green indicates the opposite.


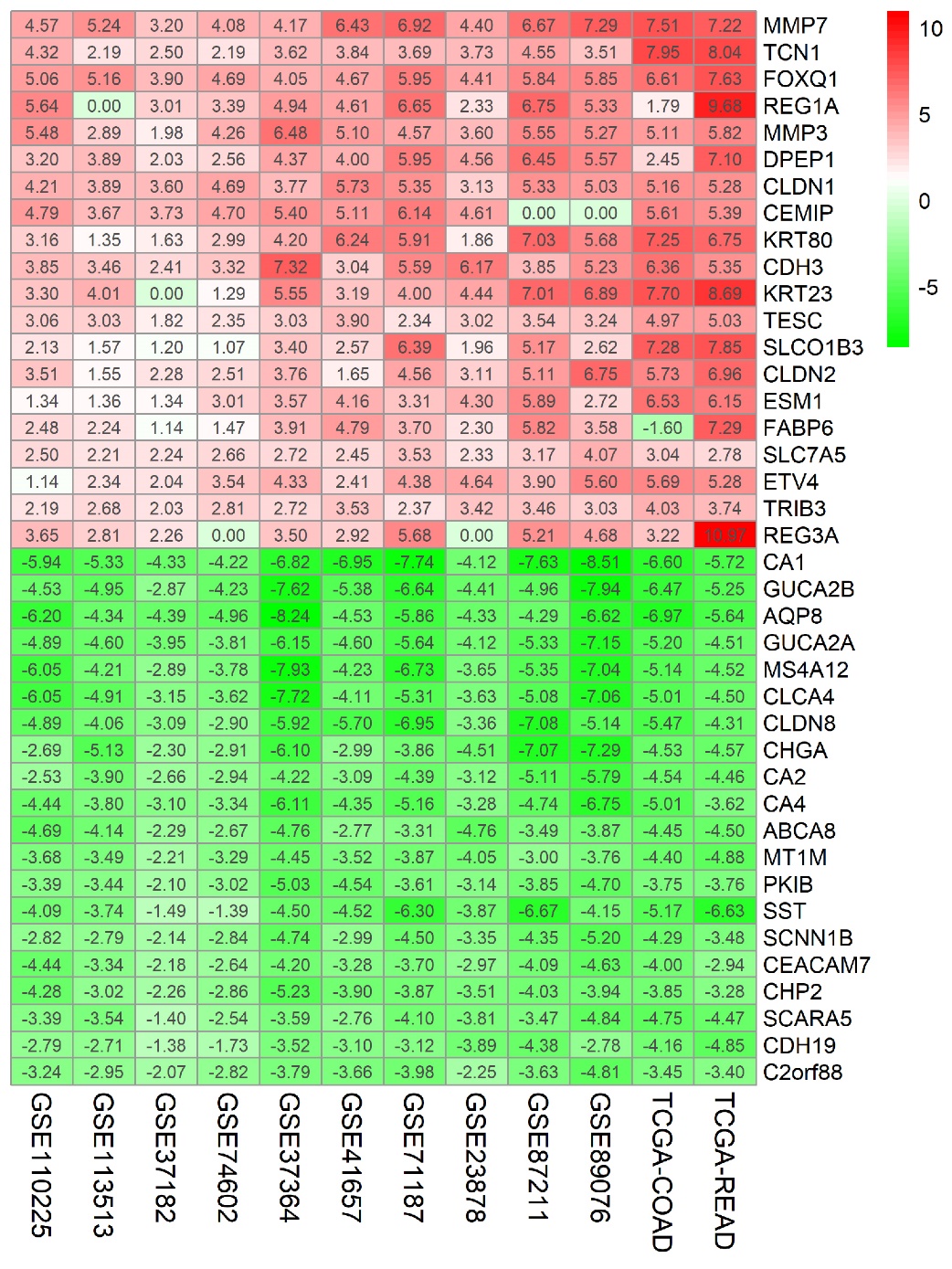


**Supplementary Figure. S4:** Based on the logarithmic fold change value of each DEG, the top 20 up and down regulated robust DEGs analyzed in Group B (Tumor/Normal) by RRA are shown in this heatmap. The value of “0” indicates that the gene corresponding to the row is missing in the data set corresponding to the column. Red cells indicates that DEGs are upregulated in CRC samples, while green indicates the opposite.

**
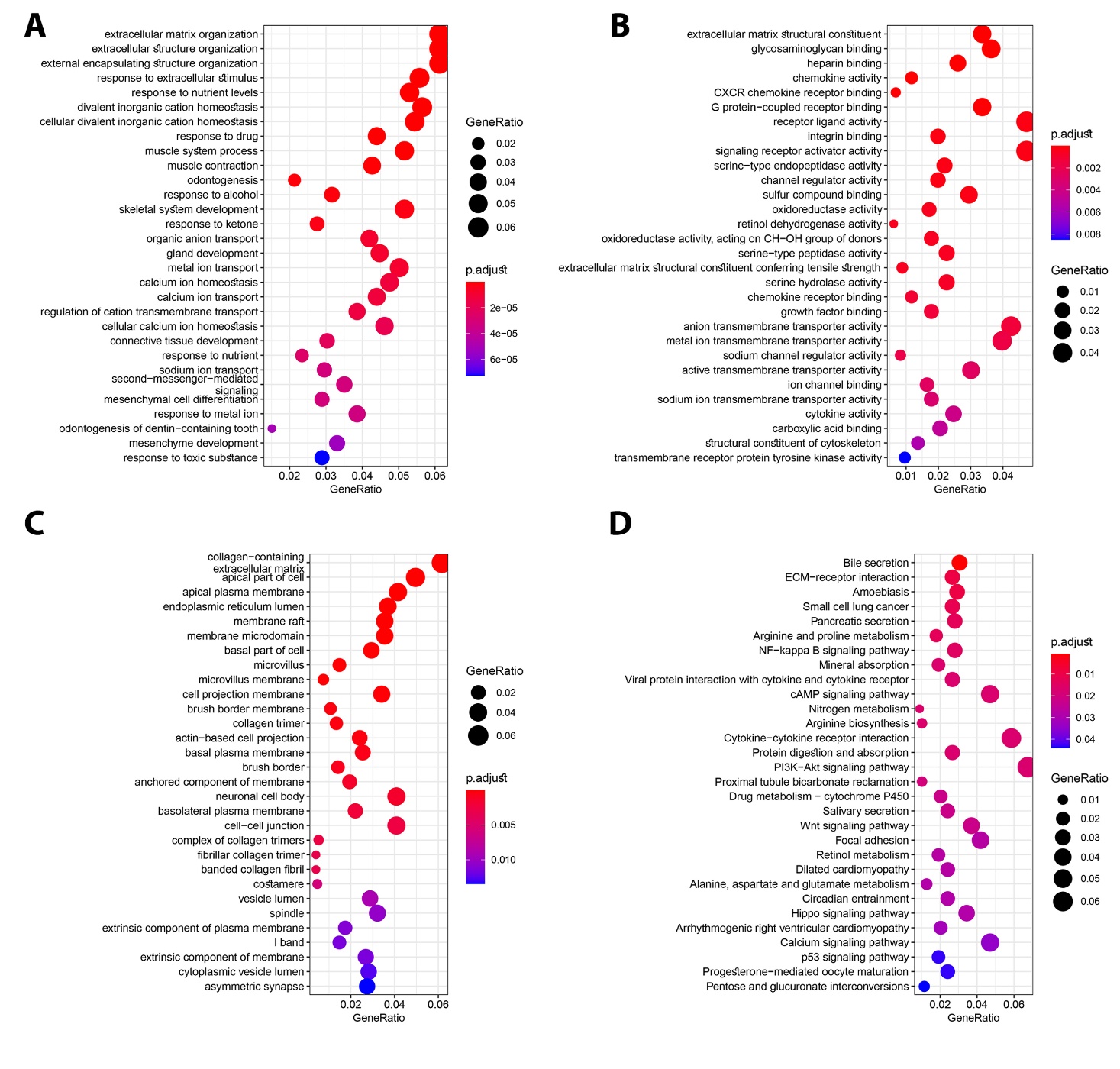
**

**Supplementary Figure. S5:** GO and KEGG pathway enrichment analyses of DEGs in all CRC stages using ClusterProfiler. (A) Results of biological process, (B) cellular component and (C) molecular function as well as (D) KEGG pathway enrichment analyses. Size of round node is in proportion to gene ratio of the enriched gene number. GO, Gene Ontology; Kyoto Encyclopedia of Genes and Genomes.

**
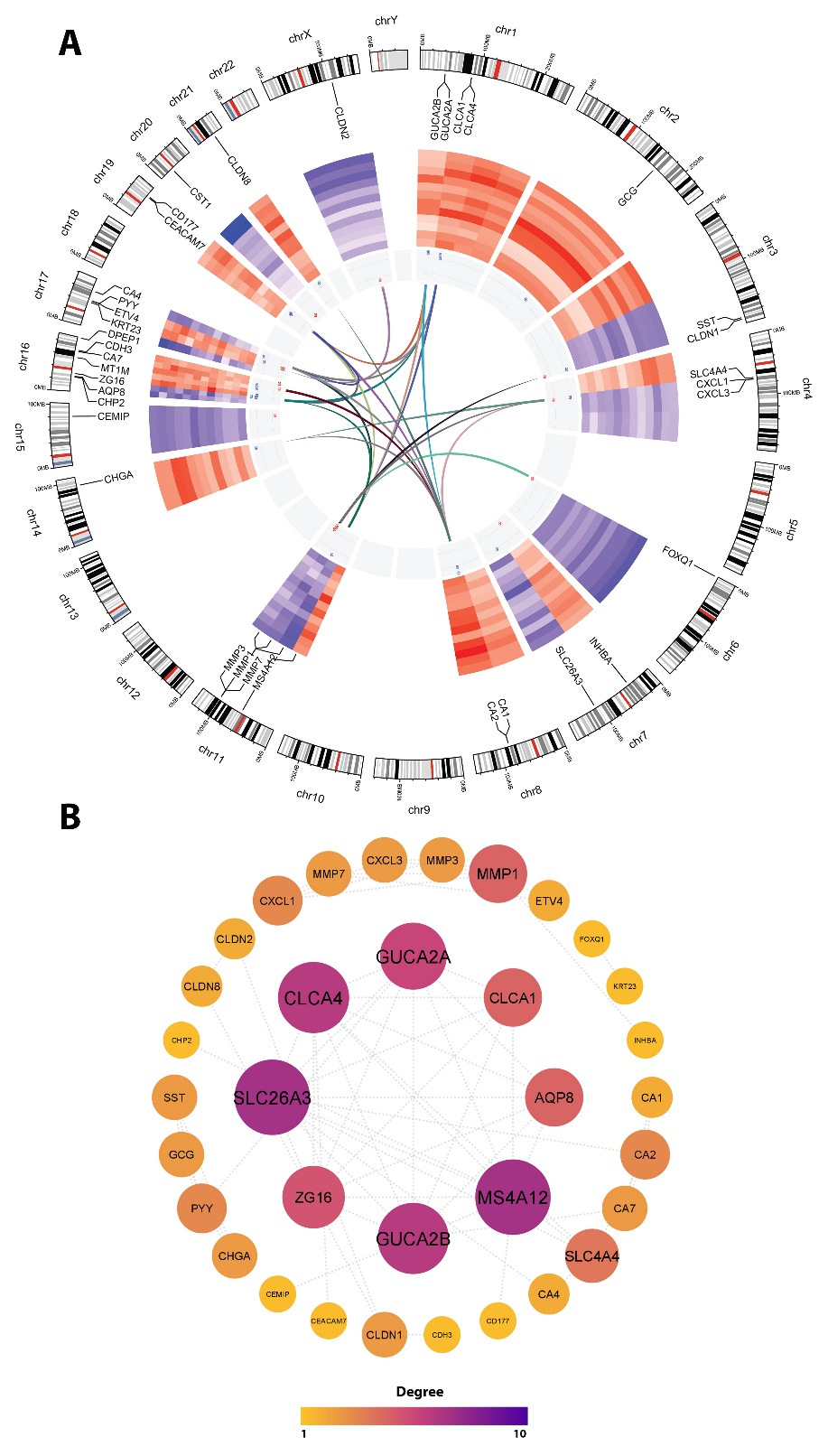
**

**Supplementary Figure. S6: (A)** Circular visualization of chromosomal locations, expression patterns, and correlation of primary hub genes from the outside to the inside. The 12 CRC microarray and RNA-seq datasets are displayed as heatmaps. Red indicates gene up-regulation, blue represents downregulation in a given dataset. The top genes with the correlation coefficient of >0.85 are shown in the center of circle. **(B)** The most important hub genes with the highest degree are shown in the center of the PPI network.

**
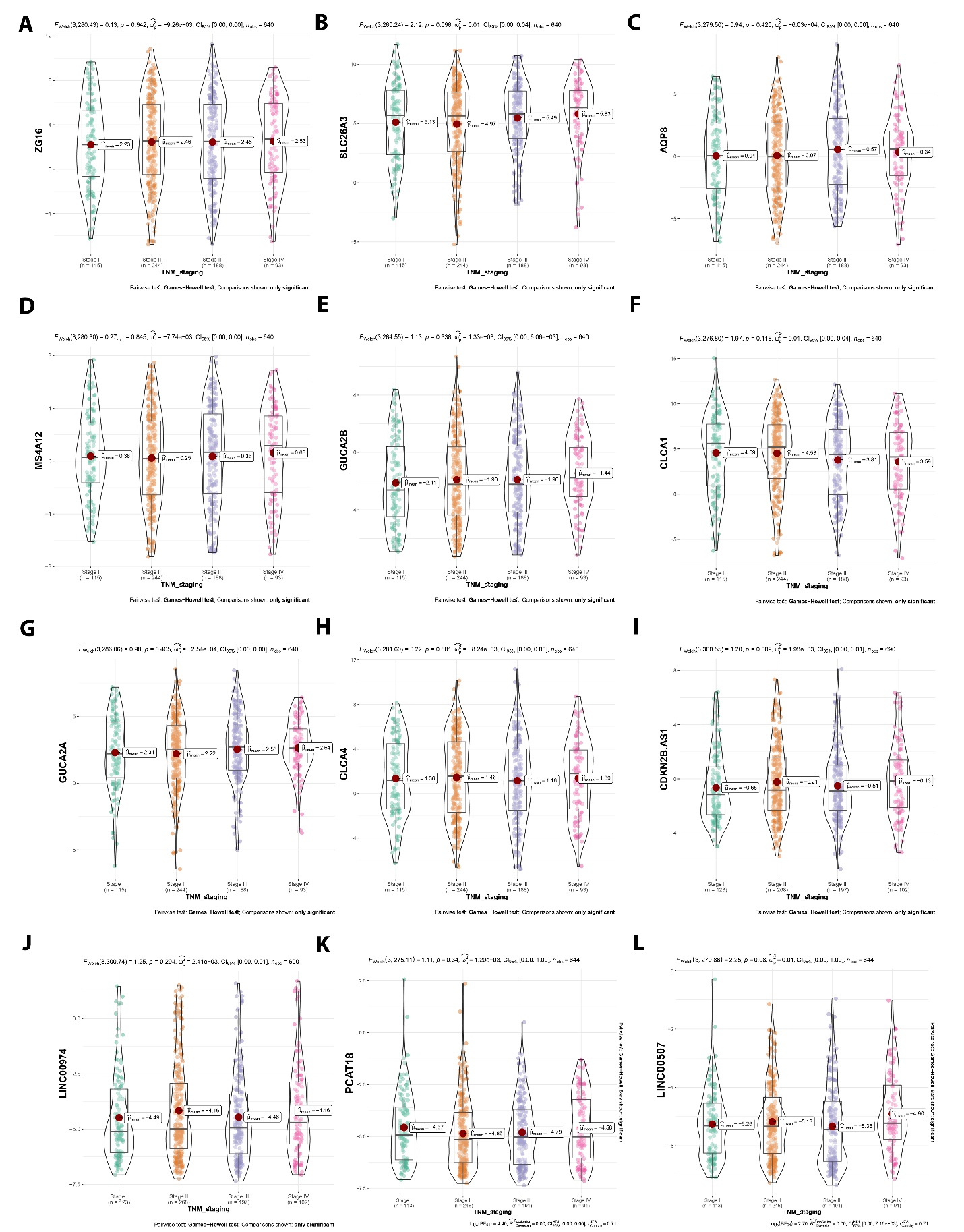
**

**Supplementary Figure. S7:** Cohort validation of final eight hub genes (mRNAs) and four-lncRNA signature from the TCGA-COADREAD database in different AJCC cancer stages (I to IV).


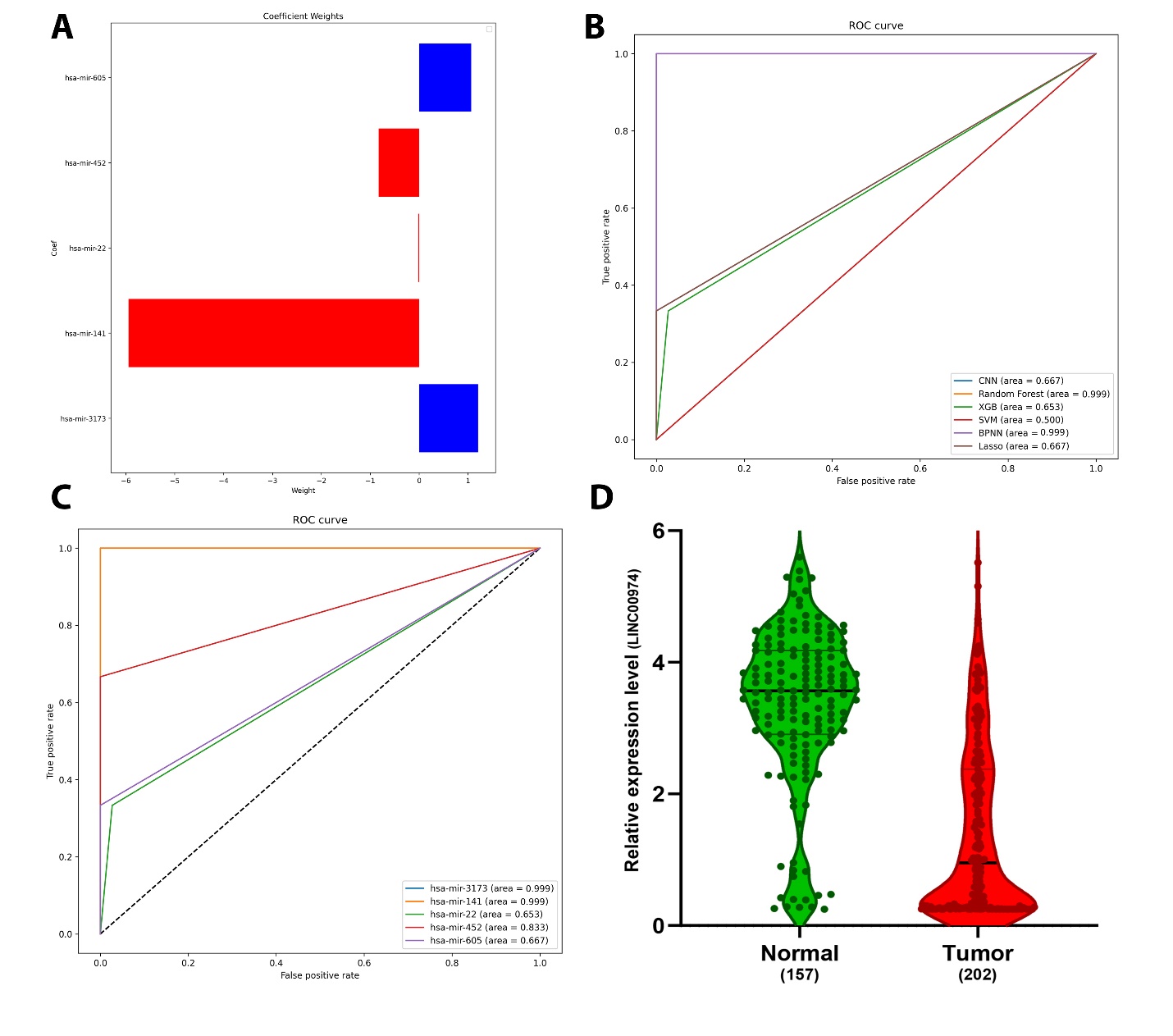


**Supplementary Figure. S8: (A)** Feature selection using LASSO. where the non-zero coefficients of 7 5 miRNAs as features was calculated and displayed. **(B)** The ROC curve showed the prediction accuracy of different six models on the test set. The AUC value of each model represents the area under the ROC curve. The closer the AUC to 1, the better the generalization of the model. **(C)** The back-propagation neural network (BPNN) provided the most efficient estimates. **(D)** Validation of the LINC00974 expression in GSE87211 GEO dataset.
